# Sensitive Method for the Confident Identification of Genetically Variant Peptides in Human Hair Keratin*

**DOI:** 10.1111/1556-4029.14229

**Published:** 2019-10-31

**Authors:** Zheng Zhang, Meghan C. Burke, William E. Wallace, Yuxue Liang, Sergey L. Sheetlin, Yuri A. Mirokhin, Dmitrii V. Tchekhovskoi, Stephen E. Stein

**Affiliations:** ^1^ Biomolecular Measurement Division Mass Spectrometry Data Center National Institute of Standards and Technology 100 Bureau Drive Gaithersburg MD 20899

**Keywords:** forensic science, genetically variant peptide, hair protein extraction, cuticular keratins, peptide mass spectral library, trace detection

## Abstract

Recent reports have demonstrated that genetically variant peptides derived from human hair shaft proteins can be used to differentiate individuals of different biogeographic origins. We report a method involving direct extraction of hair shaft proteins more sensitive than previously published methods regarding GVP detection. It involves one step for protein extraction and was found to provide reproducible results. A detailed proteomic analysis of this data is presented that led to the following four results: (i) A peptide spectral library was created and made available for download. It contains all identified peptides from this work, including GVPs that, when appropriately expanded with diverse hair‐derived peptides, can provide a routine, reliable, and sensitive means of analyzing hair digests; (ii) an analysis of artifact peptides arising from side reactions is also made using a new method for finding unexpected modifications; (iii) detailed analysis of the gel‐based method employed clearly shows the high degree of cross‐linking or protein association involved in hair digestion, with major GVPs eluting over a wide range of high molecular weights while others apparently arise from distinct non‐cross‐linked proteins; and (v) finally, we show that some of the specific GVP identifications depend on the sample preparation method.

In recent publications from Lawrence Livermore National Laboratory (LLNL), genetically variant peptides (GVPs) derived from human hair have been shown to have forensic value 1, 2. The publication 1 by Parker et al. showed that these peptides might serve as a source of evidence in addition to DNA for human identification due to several advantages that a hair sample carries: (i) commonly found—on average, humans shed 50–150 hairs per day; (ii) stable—proteins in a hair sample usually last longer and are more resistant to degradation than DNA; and (iii) when good quality DNA is not available, hair proteins may serve as alternative evidence by detecting those GVPs in hair cuticular keratins and other hair proteins. A recent publication 2 by Mason et al. described protein‐based or GVP‐based human identification from a single hair as short as 1 inch long. Another recent publication 3 by Carlson et al. described a sensitive method to extract proteins from 1 millimeter or less in total length of human anagen head hairs and compared the proteins identified from hair shaft and hair root. The effectiveness of this method for detecting GVPs has not yet been determined.

The human hair shaft is made up of three main components 4. Starting from the center, the first component is the medulla which is rich in cross‐links and highly insoluble. Next is the cortex which comprises most of the hair shaft and is made up of hair cuticular keratin fibrils as well as keratin‐associated proteins. The thin outer layer is the cuticle which is also composed of keratin‐associated proteins and is the component that would be visually inspected through microscopic examination. Hair cuticular keratins have been classified as type I (31‐38) and type II (81‐86) based on the finding that type I keratins are acidic and type II keratins are neutral or basic proteins 5, 6. Two recent publications 1, 2 from LLNL have collectively identified a total of 88 GVP sites from multiple donors with bulk of hair samples: 32 sites from hair cuticular keratins, 7 sites from cytoskeletal keratins, 22 sites from keratin‐associated proteins, and 27 sites from nonkeratins.

Based on these findings, a human hair sample has the potential to serve as alternative evidence for human identification if GVPs in hair keratins (mainly cuticular keratins), keratin‐associated proteins, and other nonkeratin hair proteins can be sensitively and reliably identified. To detect them, we first need an efficient method to extract proteins from human hair shafts. However, hair protein extraction is especially difficult due to extensive cross‐linking and poor solubility of hair keratins 7, 8, 9. In this manuscript, we describe a direct protein extraction method (referred as the direct method) that can efficiently extract hair proteins from a single hair shaft less than 1 cm in length. We performed GVP panel analyses and examined experimentally introduced artifactual modifications among three methods: our newly developed direct method and two of previously published methods—NaOH‐based SDS repeated extraction method (we modified it to make it fit in small sample analysis, referred as modified NaOH + SDS method) 8 and ProteaseMAX‐based method (referred as cleavable surfactant method) 1, 2. Considering the direct method and modified NaOH + SDS method both utilize protein gel electrophoresis to separate extracted proteins, we made further comparisons between these two in‐gel methods for sensitivity and reproducibility. We find that the direct method is both sensitive and relatively convenient to carry out while generating reproducible results regarding GVP detection from a single hair shaft from one individual donor. In the analysis of these data, we applied a number of proteomic data analysis methods including (i) the development of a library of peptide ion spectra containing all identified peptides that, when extended, can contain all identifiable peptides from hair proteins. Spectral libraries provide a sensitive and reliable means of peptide identification and ultimately can contain spectra of all known GVPs. (ii) Proteomic analysis enables the detailed analysis of artifact peptides, generated by undesirable chemical analysis which can, in principle, lead to false‐positive analysis. (iii) A gel‐based method of analysis reveals a wide distribution of molecular weights of proteins yielding keratin‐based GVPs. (iv) The finding that different digestion methods can identify different GVPs suggests the inadequacy of any current method of finding all potentially identifiable GVPs in a hair sample.

## Materials and Methods

### Human Hair Sample Preparation

Human hair samples were obtained commercially from BioreclamationIVT (LOT# BRH1363732, 5 g of hair shaft per package from the same individual donor). Most of the results presented in this manuscript are derived from hair shafts from this single randomly selected donor: Asian male, 30 years old. Hair samples were briefly washed with 20% methanol and water, then dried, and stored at −20°C. The related protocols have been reviewed and approved by National Institute of Standards and Technology (NIST) Human Subjects Review Board.

### Direct Extraction Method

Hair shaft samples (5 cm, 2.5 cm, or 1 cm) were cut using sterile laboratory scissors and then combined with 50 µl of the commercially obtained NuPAGE lithium dodecyl sulfate (LDS) sample buffer (Catalog # NP0007; Thermo Fisher Scientific, Waltham, MA) and 50 mmol/L reducing agent dithiothreitol (DTT). After heating the hair shaft in sample buffer at 90 ^◦^C for various lengths of time, extracted hair proteins (we call this the direct method) were loaded onto NuPAGE 4‐12% Bis‐Tris protein gels (Catalog # NP0321; Thermo Fisher Scientific) and then separated by size together with a molecular weight (MW) standard (MW std) using sodium dodecyl sulfate–polyacrylamide gel electrophoresis (SDS‐PAGE) at 200 V for 30 min. The protein gel was stained with SimplyBlue SafeStain (Catalog # LC6060; Thermo Fisher Scientific) for one hour. After overnight immersion in water, the destained‐protein‐containing gel was scanned, and intensities of the main bands were determined. From top to bottom, the gel was evenly cut into 10 fractions (about 4 mm long per fraction) and in‐gel digestion was performed for each fraction by following a well‐established in‐gel digestion protocol 10. Peptide concentrations were measured by a kit provided by Pierce (Quantitative Colorimetric Peptide Assay Kit, Catalog # 23275) after desalting by ZipTip (Catalog # ZTC18S960; EMD Millipore Corporation, Burlington, MA). Desalted peptides were injected into a Thermo Orbitrap Fusion™ Lumos™ Tribrid™ Mass Spectrometer for liquid chromatography–tandem mass spectrometry (LC‐MS/MS) analysis. A simplified direct method workflow is shown in Appendix S1.

**Figure 1:**
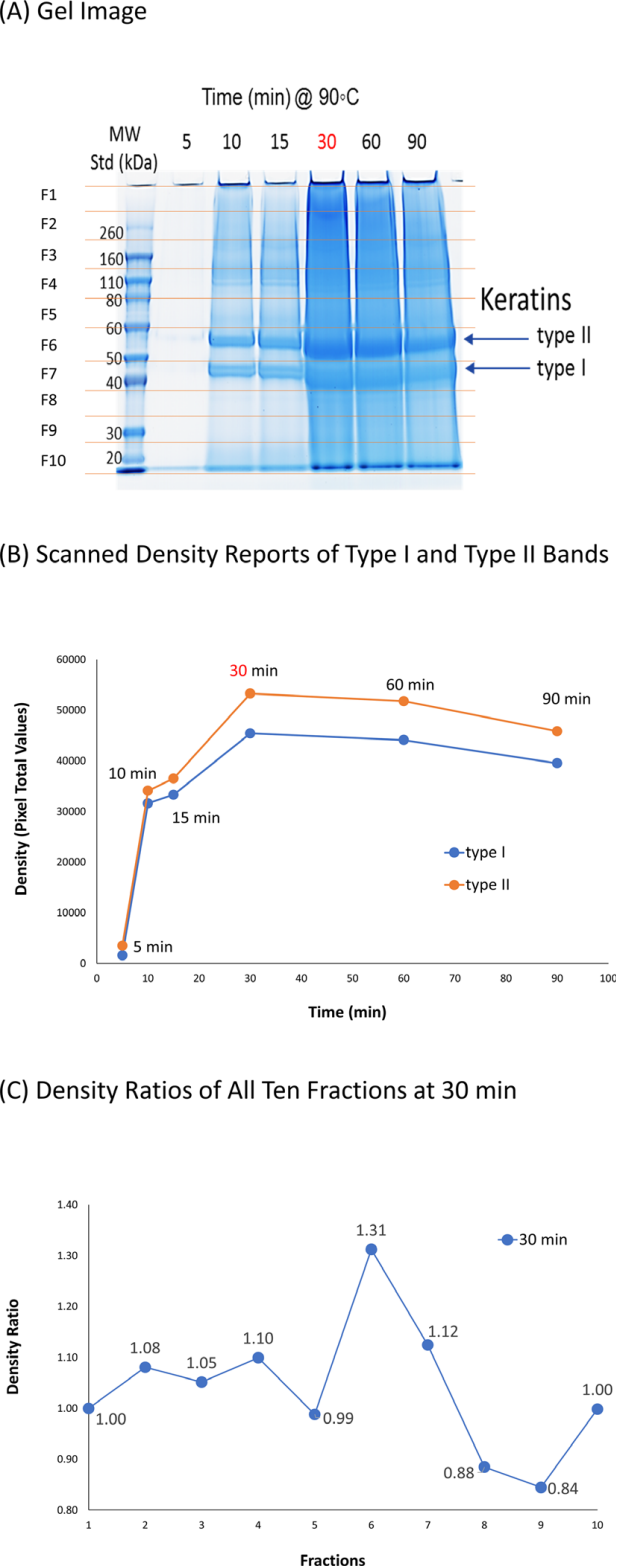
Time‐course study to optimize the best heating condition of the direct method. A time‐course study was performed to find the optimal time that a 5‐cm hair shaft sample need to be heated at 90°C. (A) The scanned gel image included a MW standard loaded in the first lane and six additional lanes where the samples were loaded on increasing length of time for which they have been heated at 90^◦^C (5, 10, 15, 30, 60, and 90 min). The major bands that correspond to type I and type II hair cuticular keratins were labeled. The orange thin lines indicate fractionating the gel to 10 slices from top to bottom as “F1” to “F10.” (B) The chart shows the density reports of type I and type II bands at each time interval. The density reports were obtained from gel scanning. The best time point (30 min) is labeled in red based on giving the maximum density reports for both type I and type II bands at 30 min. (C) The chart shows the density ratios of all 10 gel fractions obtained at 30 min, using fraction 1 as the reference.

We performed a time‐course study to determine the optimal heating time for extracting hair proteins by this direct method using six individual 5‐cm‐long hair shafts with each one processed at a different incubation time in the same amount of sample buffer (Fig. 1). The six different incubation times were as follows: 5, 10, 15, 30, 60, and 90 min with net peptide yields measured by combining all ten fractions. The largest yield of peptides was found to occur at 30 minutes and was selected as the optimal incubation time. Note that the LDS sample buffer was unchanged at a pH of 8.5 through all incubation times. As Fig. 1A shows, we observed two distinct bands: The first was found to be enriched in type II (basic) hair cuticular keratins (gene name: KRT81 to 86, # amino acids: 486 to 600, MW 53.5 to 64.8), and the second enriched in type I (acidic) hair cuticular keratins (gene name: KRT31 to 38, # amino acids: 404 to 467, MW 45.9 to 52.2) 8. The orange thin lines in Fig. 1A also indicate an even fractionation of the gel in 10 slices per lane from top to bottom as F1 to F10. Fraction 6 (F6) contains the first main band which enriches type II cuticular keratins, and fraction 7 (F7) contains the second main band which enriches type I cuticular keratins (discussion of this observation can be found in the Results and Discussion section). Figure 1B shows the density reports of type I and type II bands at each time interval, reaching a maximum at 30 min (Fig. 1B), consistent with the time for maximum peptide yield described above. Figure 1C shows the density ratios of all ten fractions obtained at 30 min, using F1 as the reference. The maximum is at F6, which is used as a keratin‐enriched representative fraction. Figure 1C indicates that the gel‐based method both concentrates known GVP‐rich keratin proteins and shows the hitherto unknown distribution of apparently cross‐linked proteins.

We note that additional studies are needed to understand both the effect of heating and the influence of cysteine alkylation and other chemical processing details on peptide yields.

### Modified NaOH‐based SDS Repeated Extraction Method

To examine our newly developed direct method, we compared it to a previously published NaOH‐based SDS repeated extraction method 8. We modified the published protocol to fit the purpose of protein extraction from a single hair shaft. The modified workflow was performed as follows (also illustrated in Appendix S1): (i) First, we used bead milling for sample preparation instead of incubation with lysis buffer: 5‐cm‐long hair shafts are ground by a bead mill (Omni Bead Ruptor 24 Elite; Omni International Inc., Kennesaw, GA) repeatedly (3 cycles, 30‐second grinding at the speed of 5 m/s and 30‐second dwell); (ii) next, ground hair samples are incubated with a NaOH‐based lysis buffer that contains SDS and beta‐mercaptoethanol (ßME) for three cycles according to published 8 protocol, and in each cycle, the hair residue is recycled through the process with bead milling; (iii) pooled supernatant containing hair proteins are precipitated with acetone; (iv) pellets from protein precipitation and leftover hair debris are combined for downstream SDS‐PAGE; and (v) in‐gel digestion was used to generate peptides.

### Hair Peptide Mass Spectral Library Construction Including Published GVPs

Using the mass spectral library construction pipeline described in the literature 11, the raw mass spectral data files generated in the present studies were used to construct a hair‐specific peptide mass spectral library. This relatively small library contains 6280 spectra (6280 peptide ions of 4343 distinct peptides, higher‐energy collisional dissociation (HCD) = 30eV), and among these—a total of 3754 spectra (3754 peptide ions of 2240 distinct peptides, HCD = 30eV) arose from hair keratins or keratin‐associated proteins—using the National Center for Biotechnology Information (NCBI, downloaded March 2017) human protein FASTA file with 20,183 sequences plus additional 51 published GVP sequences 1. This provides a sequence coverage of hair cuticular keratins of about 70%. Of these spectra, 40 mass spectra are identified as GVP ions which cover 14 published GVP sites (a subset of total 88 published GVPs): 10 sites from hair cuticular keratins, 1 site from a keratin‐associated protein, and 3 sites from nonkeratin proteins. Detailed information can be found in the Results and Discussion section where we discuss GVP panel analysis.

### Spectrum Library Searching

Freely available MSPepSearch software (peptide.nist.gov) 11 was used to perform mass spectral library searching using a precursor ion tolerance of 20 ppm (ppm was defined as parts per million) and fragment ion tolerance of 50 ppm. Label‐free HCD human tryptic peptide spectral libraries (version September 23, 2016, contains 1,127,970 spectra, indicated as “main” library) are available online (peptide.nist.gov) 12. A hair‐specific peptide spectral library (indicated as “hair” library) 13 was created from 90 raw mass spectral data files generated during method development of processing 16 five‐cm‐long hair shafts of this same individual Asian donor. Surprisingly, 40% of peptides contained in this “hair” library were not present in the “main” library even though it was constructed from a wide range of publicly available data files. Clearly, hair was not a common protein‐containing material in these studies. This “hair” library was used in combination with the “main” library for mass spectrum library searching. The 1% false discovery rate (FDR) level was determined by using the target‐decoy method described in the literature 14, 15. The NIST‐formatted mass spectral libraries were built using the program Lib2NIST freely available online at chemdata.nist.gov. This library and associated software are freely available online 13.

### Sequence Database Searching

We used the Sequest 16 HT search node implemented in Proteome Discoverer (PD) 2.1 for initial peptide identification prior to entry into a library and comparison of the results of spectral library searching. Mass tolerance settings were the same as in the library searches. The top scoring peptide identification was selected, and FDR level was set at 1% using the same FASTA file described above.

### Proteomic Methods

GVP and its nonvariant form designation: In this work, GVPs are tryptic peptides that are represented first by their gene name followed by the site of the amino acid substitution. For example, “DSP R1783Q_Q” indicates the tryptic peptide derived from desmoplakin (GN = GSP) containing “Q” at position 1783. The corresponding nonvariant form is “DSP R1738Q_R” where “R” is in place of “Q.” The term “GVP ion” refers to not only tryptic peptide sequence, but also charge state and possible modifications. Peptides observed in different charge states or modifications are treated as different peptide ions. The most abundant form of a peptide ion is used to measure its intensity.

LC‐MS/MS parameters: Digests were analyzed on an Eksigent Classic 2D Nano LC with an Acclaim PepMap RSLC column (75 µm × 15 cm, C18, 2 µm, 100 Å) with a nanospray source connected to a Thermo Orbitrap Fusion™ Lumos™ Tribrid™ Mass Spectrometer in the positive ion mode. Mobile phase A consisted of 0.1% formic acid in water, and mobile phase B consisted of 0.1% formic acid in Acetonitrile. The peptides were eluted by increasing mobile phase B from 1% to 90% over 200 min. Data were collected using a data‐dependent mode with a dynamic exclusion of 20 s. The top 10 most abundant precursor ions were selected from a 350‐1600 m/z full scan for fragmentation. The resolution of full MS scan was set at 120,000, and the resolution of MS/MS scan was set at 30,000. In future work, we plan to perform a 2D‐LC study to find more trace ions.

Modifications included in hair library are as follows: (i) fixed carbamidomethyl (CAM) at cysteine (C); (ii) oxidation at methionine (M); (iii) acetylation (acetyl) at peptide N‐terminus; (iv) acetaldehyde at peptide N‐terminus; (v) Gln‐>pyro‐Glu at glutamine (Q) at peptide N‐terminus; and (vi) Glu‐>pyro‐Glu at glutamic acid (E) at peptide N‐terminus. Other less abundant modifications may be added to future versions of the library, although these may be depended on the specific chemical processing involved in the digestion.

**Figure 2:**
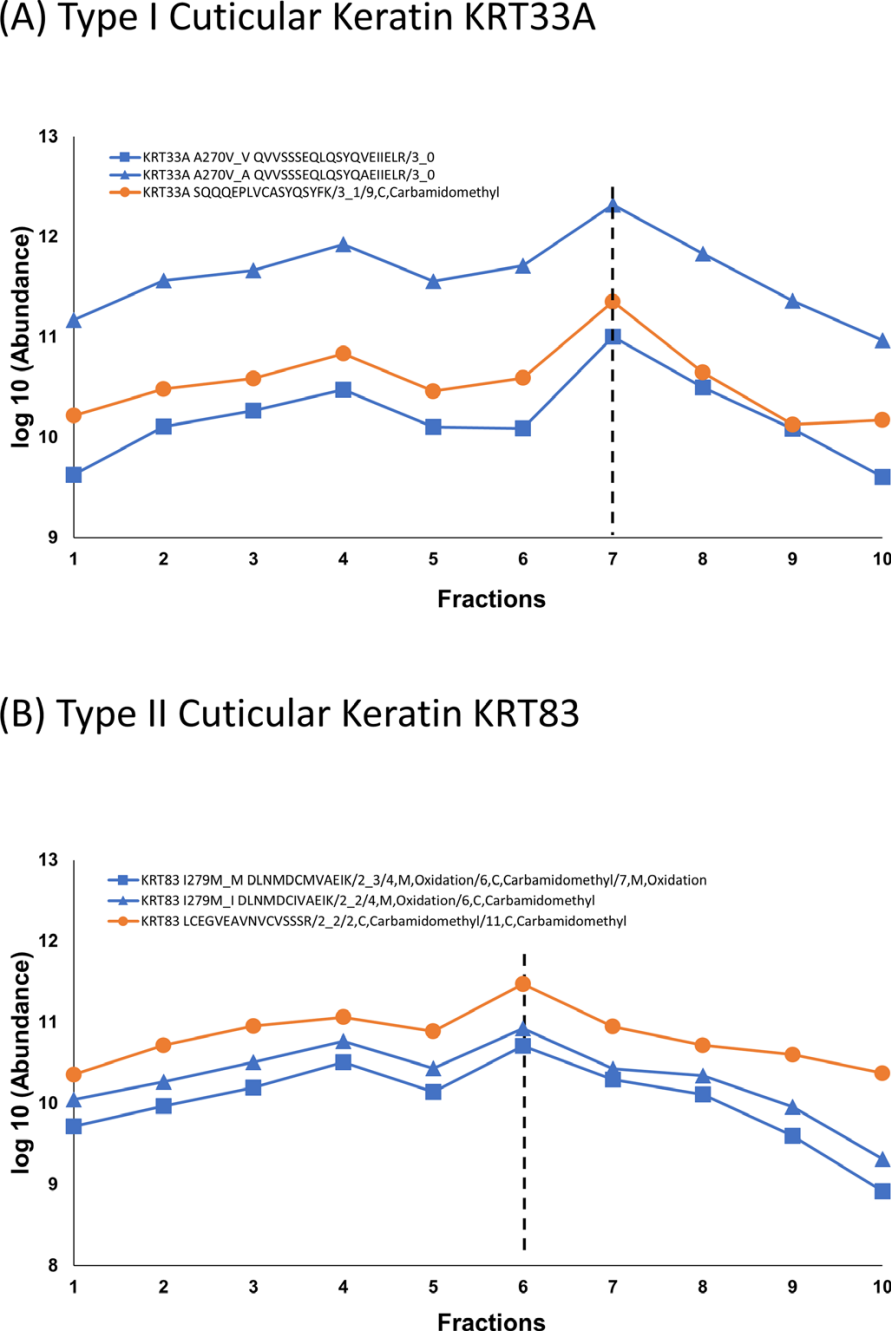
The range of the intensities of example peptide ions across all ten fractions from the direct method in type I and type II cuticular keratins. (A) Type I cuticular keratin KRT33A: the range of intensities of an example GVP peptide ion pair (KRT33A A270V_V: QVVSSSEQLQSYQ[V]EIIELR/3_0 (blue square linked by blue line) and KRT33A A270V_A: QVVSSSEQLQSYQ[A]EIIELR/3_0 (blue triangle linked by blue line)) as well as another peptide ion (SQQQEPLVCASYQSYFK/3_1/9, C, Carbamidomethyl (orange circle linked by orange line)) whose sequence is unique to KRT33A but not containing a known GVP site across all 10 fractions. “KRT33A A270V_A” or “KRT33A A270V_V” means the amino acid at position 270 of KRT33A can be a “A” (regular version in human FASTA file) or a “V” (published variable version). Dashed black line indicates these three peptide ions reach their maximum intensities at fraction 7. (B) Type II cuticular keratin KRT83: the range of intensities of an example GVP peptide ion pair (KRT83 I279M_M DLNMDC[M]VAEIK/2_3/4,M,Oxidation/6,C,Carbamidomethyl/7,M,Oxidation (blue square linked by blue line) and KRT83 I279M_I DLNMDC[I]VAEIK/2_2/4,M,Oxidation/6,C,Carbamidomethyl (blue triangle linked by blue line)) as well as another peptide ion (LCEGVEAVNVCVSSSR/2_2/2,C,Carbamidomethyl/11,C,Carbamidomethyl (orange circle linked by orange line)) whose sequence is unique to KRT83 but not containing a known GVP site across all 10 fractions. “KRT83 I279M_I” or “KRT83 I279M_M” means the amino acid at position 279 of KRT83 can be an “I” (regular version in human FASTA file) or a “M” (published variable version). Dashed black line indicates these three peptide ions reach their maximum intensities at fraction 6.

Incomplete digestion in proteomics: The inability to digest substantial portions of the proteome is common for the proteomics of biological material. Here are some examples: (i) In reference 8, the reference for the original NaOH + SDS method, hair pellets were simply discarded after incubation with lysis buffer containing NaOH + SDS; (ii) in reference 9, scanning electron microscope images as Fig. 2 to show remaining undigested hair after extraction with SDS or with urea. In cases 1 and 2, substantial portions of the hair undigested although it is method‐dependent; (iii) in reference 17, heavy‐isotope‐labeled proteins were used to compare peptide recovery between laboratories and the results showed that the digestion step was the greatest source of inconsistent recovery (median loss of 70%). These examples demonstrate that significant levels of incomplete digestion are expected in the proteomics of biological materials.

## Results and Discussion

### Identification of Hair Proteome including Cuticular Keratins by Direct Extraction Method

We examined overall protein and peptide identifications from all ten gel fractions and compared our library search results to the results from sequence (Sequest) searches. When searching spectral libraries, we added the “hair”‐specific mass spectral library to our “main” library 12, 13 to obtain better search performance. The next A and B subsections discuss these results and demonstrate the effectiveness of spectral library searching for peptide identification. In subsection C, we examine GVP detection with library searching in all ten fractions and compare the GVP panel analysis by the direct method to the other two published methods 1, 8.

#### Overall Gel Identification

Results for hair proteins extracted from a single 5 cm long hair by the direct method are presented in Table 1. They were derived from one raw MS data file for each of the ten gel fractions. All were independently analyzed to determine details of the gel separation and digestion process.

**Table 1:** Comparison of protein and peptide identifications from spectral library and Sequest searching in all ten fractions at 1% FDR by the direct method from a 5‐cm‐long hair shaft.*

Direct	Yield (µg)	TIC	Main + Hair Spectral Library				Sequest			
			Hair Proteome		Cuticular Keratins		Hair Proteome		Cuticular Keratins	
			Proteins	Peptides	Proteins	Peptides	Proteins	Peptides	Proteins	Peptides
F1	1.76	3.91E + 06	148	2040	14	583	98	1128	14	471
F2	3.81	6.54E + 06	140	1888	15	614	84	1052	14	503
F3	5.46	1.03E + 07	132	1744	14	614	73	1022	14	525
F4	8.95	1.44E + 07	134	1789	14	628	83	1045	13	526
F5	5.86	8.27E + 06	152	1781	14	594	93	1061	14	513
F6	13.25	2.06E + 07	135	1617	15	620	68	906	15	503
F7	10.92	2.31E + 07	146	1607	13	623	76	933	14	538
F8	7.06	8.17E + 06	207	2167	15	631	129	1290	15	521
F9	5.98	4.72E + 06	214	2268	14	589	138	1346	13	463
F10	12.24	8.59E + 06	173	1744	14	470	120	1079	13	347

* Proteins were identified by ≥ 2 peptides throughout this manuscript. For peptide/protein identifications (IDs) under “Hair Proteome,” fraction 8 (F8) and fraction 9 (F9) gave more IDs in both spectral library and Sequest searches; for peptide/protein IDs under “Cuticular Keratins,” the distribution of IDs was more even across all 10 gel fractions in both spectral library and Sequest searches. TIC: an index of total ion current.

Using both spectral library and Sequest searching methods, the results derived from F1 to F10 are compared in Table 1. As shown in Table 1, when the “main” library was combined with the “hair” library for spectral library searching, the overall library identification for proteins—for both hair proteome 7, 9 and hair cuticular keratins (a major subset of the hair proteome) 1, 8—was similar to that from Sequest; however, for all peptides identified, the spectral library method was somewhat more sensitive at a given FDR level, consistent with previous observations 14.

Hair cuticular keratins are major components of hair proteome. Table 2 examined the sequence coverage of listed total 15 hair cuticular keratins of type I and type II by library and Sequest searches from all ten fractions. Peptides present in multiple proteins were used in calculating the sequence coverage of each protein. Since we are interested in GVPs, of course the better coverage, the greater the chance of detecting potential GVP sites. In general, library searching provides a fuller coverage than database searching, although except for the most abundant KRT31, some of these coverages are far less than 100%. There are several possible reasons for this: (i) Cross‐linking makes certain sites hard to reach by trypsin during the digestion; (ii) extremely long (>50) or short (<6) peptides were not considered under the current search parameters; (iii) loss of extremely hydrophilic or hydrophobic peptides occurs during sample preparation and LC analysis. (iv) Incomplete conversion of proteins to peptides is common throughout proteomics, and according to reference 18, an approximately 70–80% of recovery is expected after extraction from the gel. Putting all ten fractions together, 8 out of 15 hair cuticular keratins reach more than 90% coverage, 5 out of the rest 7 reach more than 50%, and only 2 less than 50% (KRT37 and KRT84). Appendix S2 shows sequence coverage in amino acids of 15 type I and type II hair cuticular keratins found by library and Sequest searches.

**Table 2:** Comparison of sequence coverage (%) of hair cuticular keratins from spectral library and Sequest searching in all ten fractions by the direct method.

Cuticular Keratins	From Library	From Sequest
KRT31	100.0	97.6
KRT32	54.2	49.6
KRT33A	97.0	93.3
KRT33B	97.0	93.6
KRT34	86.0	83.9
KRT35	91.0	86.4
KRT36	60.8	49.3
KRT37	43.0	34.7
KRT38	61.2	51.3
KRT81	96.2	91.9
KRT82	63.4	49.9
KRT83	97.0	87.2
KRT84	12.7	11.2
KRT85	96.8	89.4
KRT86	99.2	92.4
Average	77.0	70.8

#### Major and Minor Gel Band Identification

We observed two distinct gel bands in fractions 6 and 7 (Fig. 1). The other fractions had several minor bands, but most of the intensity was evenly distributed (Fig. 1C). Results are discussed below.

Figure 2 shows the intensities over the fractions for selected peptides from type I (A) or type II (B) hair cuticular keratin. In both cases, both the GVP and nonvariant form are shown along with another major peptide from each protein. The abundance of each peptide derived from its MS1 ion chromatogram peak area. These results indicate (i) the major gel bands correspond to type I (fraction 7) and type II (fraction 6) hair cuticular keratins, consistent with the literature 8 reports. Fractions 6 (type II) and 7 (type I) are enriched in individual hair cuticular keratins; (ii) it is noteworthy that most peptides identified outside the main regions were the same as those inside that region. This behavior persisted in all analyses. This is presumably due to the presence of significant quantities of cross‐linked proteins or unseparated complexes with higher molecular weight with lower mobilities as well as fragments of these proteins at lower molecular weights with higher mobilities. We find that keratin GVPs are found in virtually all gel fractions suggesting that they distributed among a wide range of cross‐linked proteins, suggests that the insoluble, cross‐linked portion of the hair protein may not contain additional keratin‐GVP identifications. According to reference 7, the insoluble, cross‐linked portion has a higher content of nonkeratin proteins and may contain additional non‐keratin‐GVP identifications. Further, we know of no way to enhance the method’s digestion effectiveness, though such an improvement would be very welcome.

**Figure 3:**
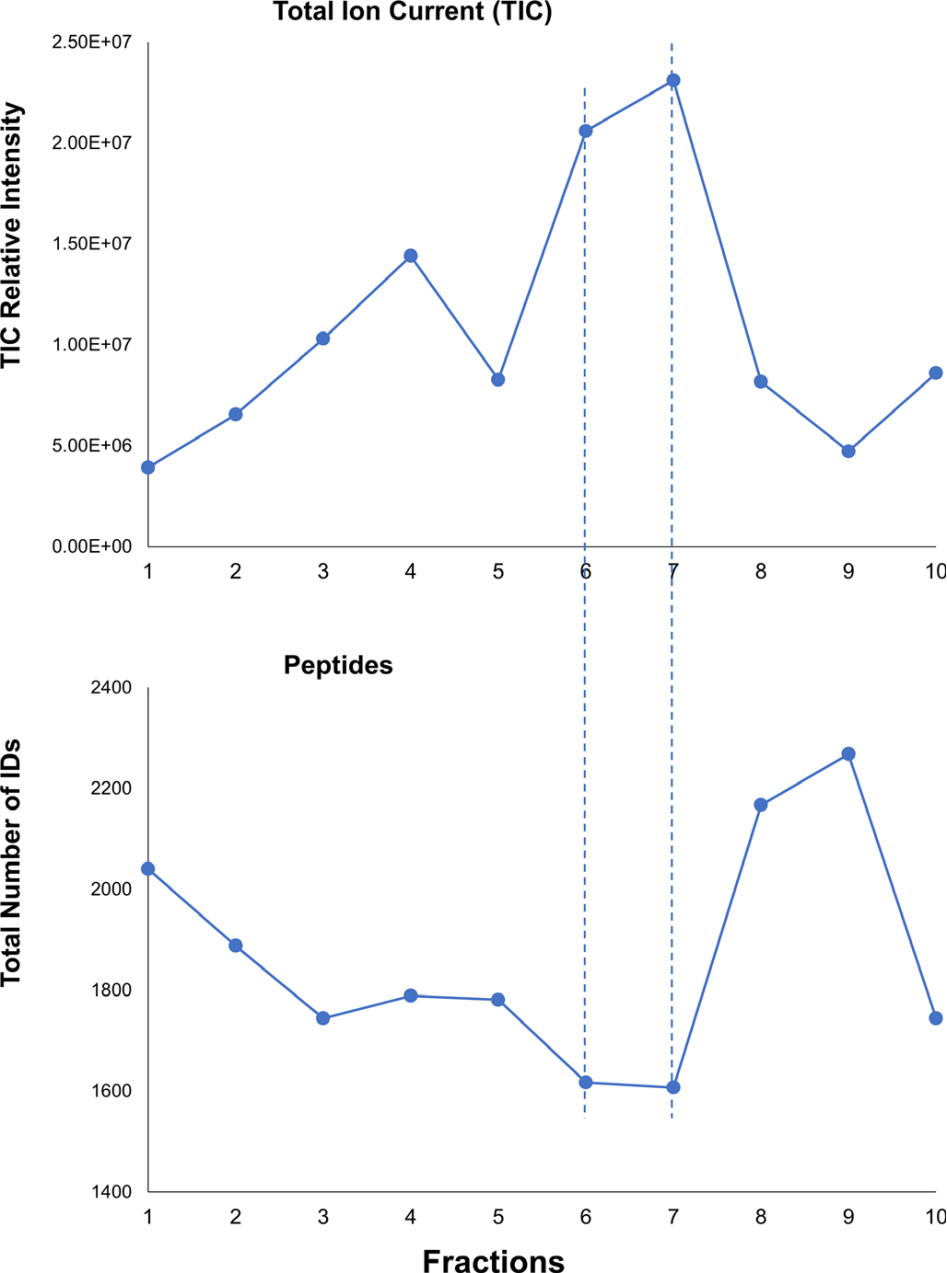
The range of total ion current (TIC, upper panel) and peptide identifications (lower panel) across all 10 fractions. Blue dashed lines indicate TIC values reach their maximum numbers at fractions 6 and 7, where peptide IDs reach their minimum numbers at fractions 6 & 7.

Note that in Table 1, fractions 6 and 7 show the highest peptide signal strengths but lowest numbers of peptide identifications (IDs). This is confirmed in Fig. 3, where the total ion currents (TICs) are inversely correlated with peptide IDs with a correlation coefficient of −0.75. This is a consequence of the higher concentrations of relatively a few proteins dominating fractions 6 (type II) and 7 (type I), which leads to higher concentrations of their tryptic peptides with consequent signal suppression of peptides from other, less abundant proteins. In other fractions, no individual proteins dominate, so tryptic peptides are more equally spread across a larger number of proteins, though many of them are cross‐linked, fragmented, or otherwise modified. Table S1 shows when moving along the gel fractions from F1 to F10, the example big protein (Desmoplakin) decreases and the example small protein (a keratin‐associated protein) increases.

The major advantage of gel fractioning is that it separates the proteins by molecular weight, thereby showing more clearly the origin in individual GVPs. It can also minimize ion suppression leading to the identification of additional GVPs. Unfortunately, this approach is time‐consuming. Our attempts to combine fractions led to the loss of potential GVPs (see section C). Identifications of all GVPs in a single‐digest analysis are apparently not possible at present (discussed below). Finding optimal methods will be the topic of future research.

#### GVP Panel Analyses in All Ten Fractions and Among Three Methods

As described in the Method section, we identified a total of 14 published tryptic GVP sites from this Asian donor’s hair samples. These sequences along with corresponding nonvariant sequences are listed in Appendix S3. Table 3 shows the specific GVP identification for the three methods with three replicate runs for each method, namely our direct method, the modified NaOH + SDS method 8, and the cleavable surfactant method 1, 2. For both the direct method and modified NaOH + SDS method, GVP panel results from different fractions are combined in Table 3. Appendix S3 uses the results from F1 to F10 as an example to illustrate how we performed this analysis for a complete data set by the direct method. Analysis led to a number of general findings:

1. For high‐abundance GVPs from major keratins, as shown in Fig. 2A or B, identifications are easily made. Scores are high [MF: 792 ‐ 942], leading to highly confident identifications 14, retention times are reproducible (Appendix S3), and identifications are made in all gel fractions for both the GVP and its nonvariant form.
2. For low‐abundance GVPs, mostly arising from less abundant proteins, identifications can be harder to assign, possibly involving lower and variable scores. Confidence can be increased by elution in the expected gel fraction as well as the determination of its nonvariant form (sometimes, this is made more difficult if GVP site involves a tryptic cleave site at R or K). This is illustrated with two examples:
  - The GVP site “DSP_R1738Q_Q: G[Q]SEADSDKNATILELR” (mutated site highlighted in brackets) was identified in the top gel fractions (F1 and F2). This is consistent with its very large precursor protein having 2871 residues, desmoplakin (DSP). This is an example that R becomes Q, and we identified both GVP and its nonvariant form in the expected gel fractions with comparable intensity (Appendix S3).
  - Another GVP site “KRTAP10‐8_H26R_R: TYVIAASTMSVCSSDVG[R]” originates from a much smaller keratin‐associated protein (KRTAP, 259 amino acids) and was recovered from bottom gel fractions (F9 and F10). This is an example that H becomes R, and we only identified GVP but not its nonvariant form. Such discrepancy happens because these are two different peptides when GVP site involves R/K. To solve this problem, we would need to choose a different digestion enzyme. Actual release rates for peptides in a protein are not easily predicted and depend on multiple factors 19. So, it is hard to estimate the relative intensities of a GVP and its nonvariant if their lengths and possibly charge states are different.
3. The specific GVP identification depends on the experiments, with a number of different GVPs identified by the in‐gel and in‐solution digestion methods. Hence, false‐negative results appear to be a significant concern with the present methods, especially for the in‐solution method.
4. We note that the identification of both a GVP and its nonvariant will significantly increase the confidence of GVP identification. Of course, this is not possible if the source is homozygous or when the nonvariant form is not an easily detectable peptide (as may be the case where tryptic cleavage sites are different in the GVP and nonvariant form). In this work, the fact that several potential GVPs were observed (Appendix S3) but not at high confidence (low abundance or matching score) reinforces the likelihood that they are not true GVPs.

**Table 3:** Genetically variant peptide (GVP) panel analyses in three methods.*

one 5 cm hair, Asian	DSP	GSDMA	KRT31	KRT32	KRT33A	KRT33B	KRT35	KRT35	KRT81	KRT82	KRT83	KRT83	KRTAP 10‐8	TGM3
	R1738Q_Q	V128L_L	A82V_V	S222Y_Y	A270V_V	V279L_L	P443A_A	S36P_P	S13R_R	T458M_M	G362S_S	I279M_M	H26R_R	T13K_K
D_Lg_F1_to_F10_r1†	X		X		X			X	X		X	X	X	X
D_Lg_F1_to_F10_r2	X		X		X	X		X	X		X	X	X	X
D_LG_F1_to_F10_r3	X		X	X	X	X		X	X		X	X	X	X
D_Lg_Combined_r1			X		X	X		X	X			X		X
D_Lg_Combined_r2			X		X	X		X	X			X		X
D_Lg_Combined_r3			X		X			X	X			X		X
D_Sg_Combined_r1			X		X			X	X			X		X
D_Sg_Combined_r2			X		X			X	X			X		X
D_Sg_Combined_r3			X		X		X	X	X			X		X
NS_Lg_F1_to_F10_r1	X	X	X		X		X	X	X		X	X	X	X
NS_Lg_F1_to_F10_r2	X	X	X	X	X			X	X		X	X		X
NS_Lg_F1_to_F10_r3	X		X	X	X		X	X	X		X	X	X	X
CS_r1			X				X			X		X		X
CS_r2			X				X	X		X		X		X
CS_r3			X				X			X				X

* All listed GVP analyses are derived from the same Asian donor’s single 5‐cm‐long hair samples: GVP panel analyses by the direct method with all 10 fractions from a long gel (30 min run at 200 V) which have been individually processed by LC‐MS/MS and then summarized the results in one row are labeled as “D_LG_F1_TO_F10”; GVP panel analyses with combined fractions processed as a mixture from a long‐gel run by the direct method are labeled as “D_LG_COMBINED”; with combined fractions from a short‐gel run (10 min run at 200 V) are labeled as “D_SG_COMBINED”; GVP panel analyses by the modified NaOH + SDS method with all 10 fractions from a long‐gel run individually processed and then summarized are labeled as “NS_LG_F1_TO_F10”; GVP panel analyses by the cleavable surfactant method are labeled as “CS.” R1, R2, and R3 are three experiment repeats.

^†^ Results from F1 to F10 are listed in Appendix S3, used as an example to demonstrate a GVP panel analysis from this “D_LG_F1_TO_F10_R1” data set.

Fractionating in the gel methods is part of a 2D study—the first dimension is separating hair proteins based on the MW during SDS‐PAGE, and the second dimension is separating extracted peptides by the LC gradient during LC‐MS/MS. Analyzing each fraction enables very low‐abundance GVPs to be identified. It is why we detect more GVPs from the two in‐gel methods than the in‐solution method. However, we detect fewer GVPs if we combine these fractions and process as a mixture (Table 3). We also tried a brief “short‐gel” run by applying SDS‐PAGE at 200 V for only 10 min (long gel: 30 min at 200 V). We compare the GVPs between long‐gel and short‐gel runs and find that short‐gel mixture loses even more GVPs (Table 3). This can be explained by hair proteins not being effectively separated in a shorter run or possibly that SDS not being fully separated from proteins. In any case, this finding highlights the importance of both separation and sensitivity in finding all identifiable GVPs in a sample. While running 10 fractions is very time‐consuming, possible GVPs were lost (Table 3) upon combining fractions indicates that more rapid analysis using a single LC‐MS/MS run can lose less abundant GVPs. Moreover, the finding that different GVPs are found with different digestion protocols implies that no existing method can be relied on to identify all possible GVPs. Together, this clearly shows the need of future work for finding the most efficient way to maximize GVP identification.

### Comparison Between the Direct Method and modified NaOH + SDS Method

Since the direct method and modified NaOH + SDS method both use protein gel to separate hair proteins, for a direct comparison, we compared the direct method with modified NaOH + SDS method for a further sensitivity and reproducibility check in this section.

#### Sensitivity

**Figure 4:**
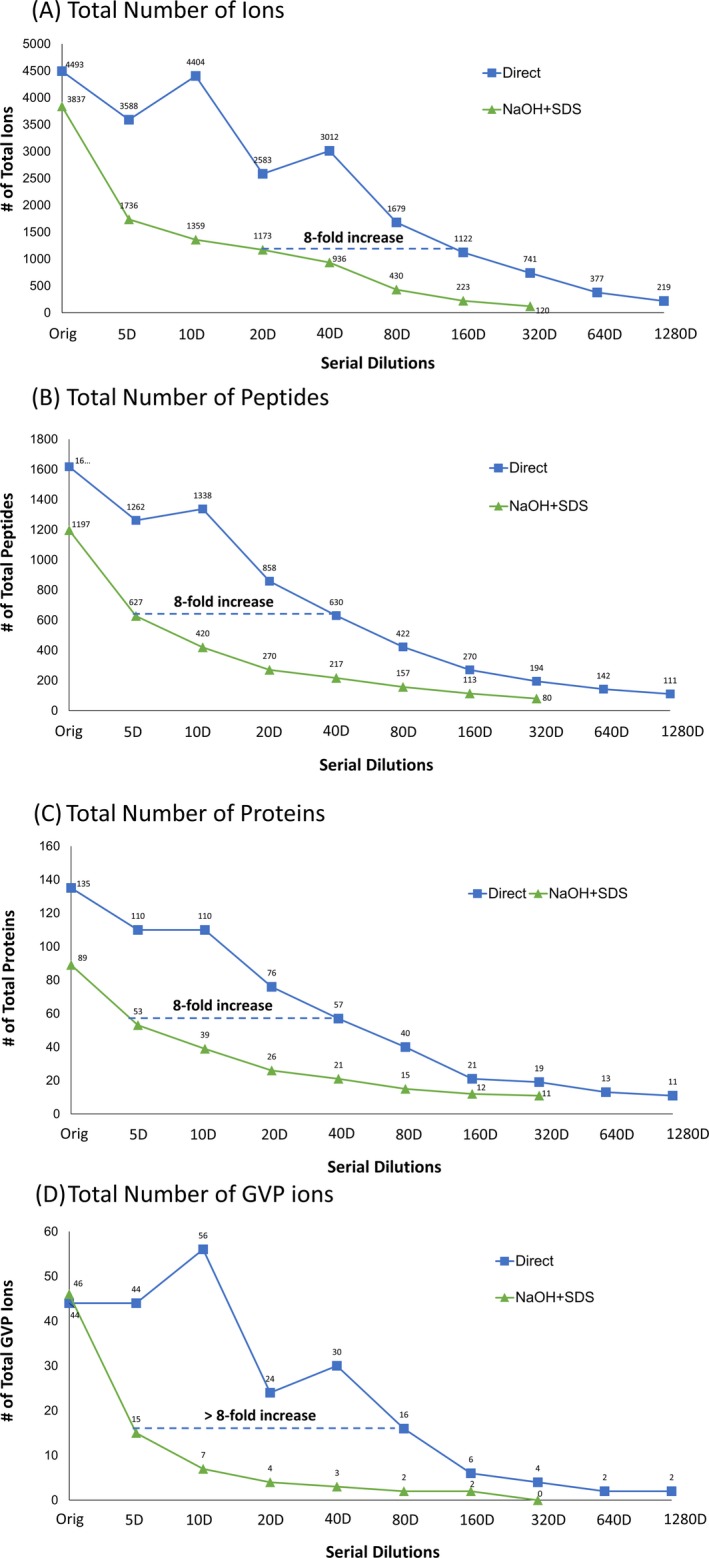
Comparison of the sensitivity in the two methods. The sensitivity of the two methods was measured by comparing multiple metrics across a dilution series from 5D to 1280D: (A) the total number of ions; (B) the total number of peptides; (C) the total number of proteins; and (D) the total number of published GVP ions detected in mass spectral data from 5‐cm‐long hair shaft sample‐derived proteins that were extracted using the direct method (blue) and modified NaOH + SDS method (green). Actual data have been labeled on the points of each dilution series.

We examine the sensitivity of the direct method to modified NaOH + SDS method by comparing multiple metrics across a dilution series. In Fig. 4, we show the relative sensitivity of the two methods by comparing the degree of dilution needed for each method to yield the similar number of IDs. After comparing total number of ions (Fig. 4A), total number of peptides (Fig. 4B), total number of proteins (Fig. 4C), and total number of GVP ions (Fig. 4D), we found that the direct method was about eight times more sensitive than modified NaOH + SDS method. The nonmonotonic behavior of some of the irregular trends is a consequence of results from the general difficulty in obtaining highly reproducible proteomic results and, for GVPs, their small numbers and therefore greater statistical fluctuation. Note that since the GVPs are few in number and variable in intensity, we could not reliably use GVPs alone to develop a reliable measure of method sensitivity based on their identifications alone. This was confirmed in a separate set of analyses: For example, GVP ions increased at 10D and then all the way decreased to minimum detection level at 1280D.

**Figure 5:**
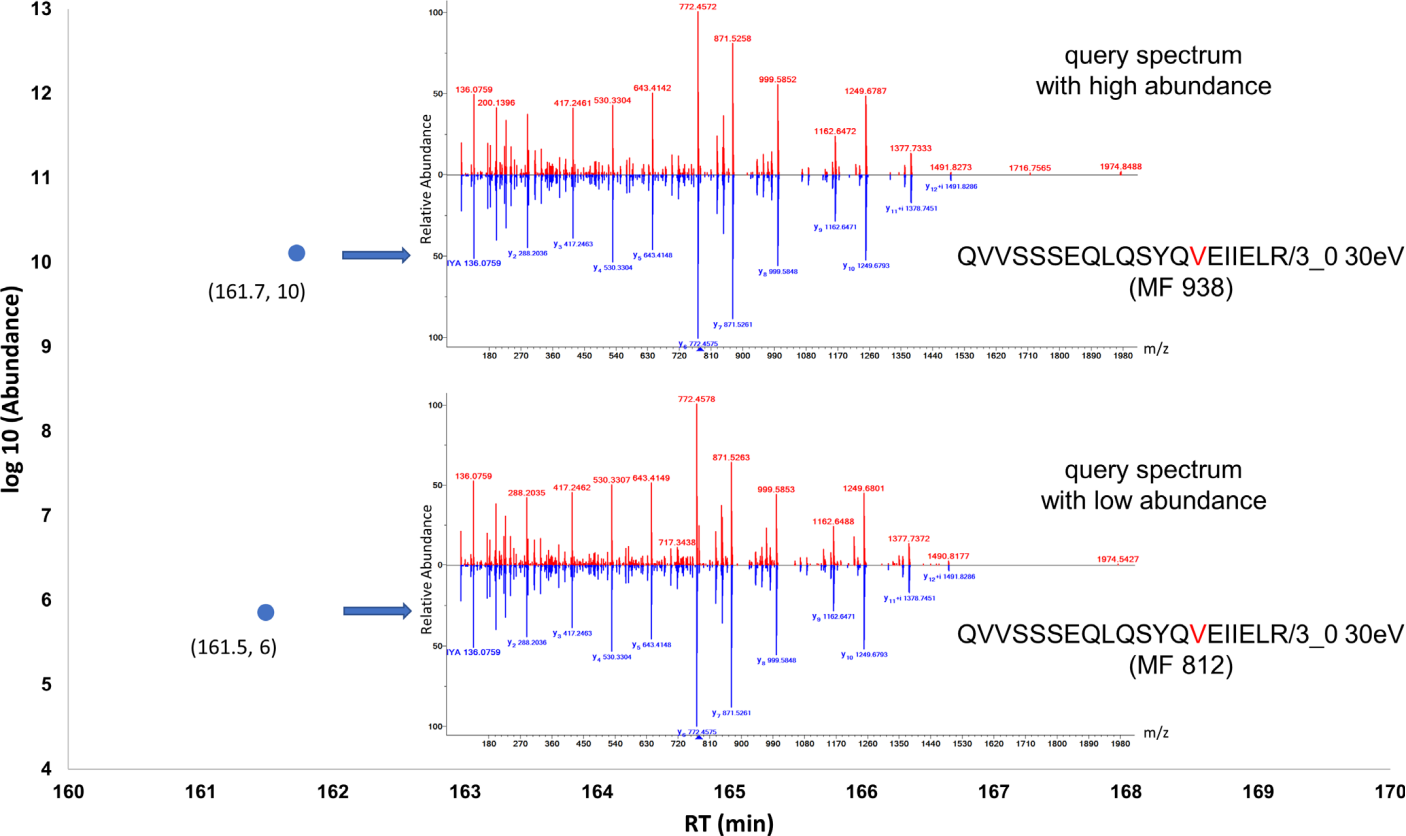
Identification of an example GVP ion with high and low abundance. The example GVP ions (KRT33A A270V_V: QVVSSSEQLQSYQ[V]EIIELR/3_0 higher‐energy collisional dissociation (HCD) =30eV) was mapped to an IonPlot (x‐axis: retention time (RT) in min, y‐axis: abundance in log 10 scale) to show the library identification with high abundance (upper blue dot) or with low abundance (lower blue dot). One blue dot indicates one peptide ion. For each blue dot, the RT and the abundance in log 10 scale were labeled underneath; blue arrows indicate their corresponding library identifications by searching the spectrum of this peptide ion as query spectrum against the hair‐specific peptide spectral library including known GVP ions. The match factor (MF) was labeled underneath its library identification.

The present direct method is both suitable for very small hair samples and able to identify GVP ions across a broad range of ion intensity. Intensities of reliably identified GVP ions could differ by orders of magnitude in ion intensity. Figure 5 illustrates this for two spectra of the same GVP ion “QVVSSSEQLQSYQ[V]EIIELR/3_0.” Even though intensities differ by four orders of magnitude, retention times were almost identical (161.7 min vs. 161.5 min) and spectral library match factors were quite high (over 800).

#### Reproducibility

**Figure 6:**
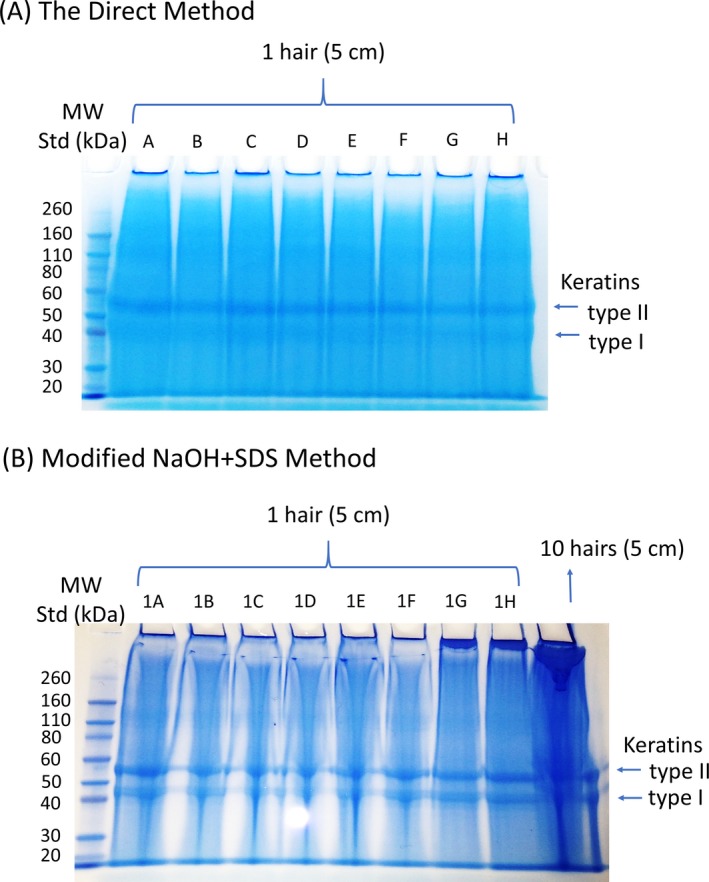
Comparison of the reproducibility of the direct and modified NaOH + SDS methods. The two gel images compare the reproducibility of methods: (A) the direct method and (B) modified NaOH + SDS method using 5‐cm‐long hair shaft samples from the same individual donor across 8 replicates (A: A to H; B: 1A to 1H). A MW standard was loaded in the first lane. Note that the NaOH + SDS gel includes a 9^t^h lane for which the extraction from ten 5‐cm‐long hair shaft samples was included as a reference. The major bands that correspond to type I and type II hair cuticular keratins were labeled.

In an examination of the reproducibility of the present method, the extraction was repeated eight times using eight individual 5‐cm‐long hair shafts (labeled as A to H in Fig. 6A) from the same donor and particularly compared it to modified NaOH + SDS method (labeled as 1A to 1H in Fig. 6B, plus the last lane from 10 hairs included as a reference). We made the assumption that each individual 5‐cm hair shaft contained the same protein mass. Figure 6 clearly indicates that the direct method is more reproducible than modified NaOH + SDS method. This presumably arises from lower sample loss for the direct method since it only needs one step/30 min for hair protein extraction, while the multiple steps (also means much longer bench time) included in modified NaOH + SDS method are more prone to sample loss and generating variable results (workflows of the two methods are shown in S1) especially when the hair sample is very small.

We also compared the protein, peptide, and GVP identifications between the direct method and modified NaOH + SDS method with analysis repeated three times for each method. Results of comparisons from a representative fraction (F6) are listed in Table 4 with three experimental repeats: (i) Higher average peptide yield (µg) was obtained in the direct method than in the modified NaOH + SDS method (11.5 vs. 2.9 µg); (ii) more average peptides were identified by the direct method than by the modified NaOH + SDS method (610 vs. 509); (iii) although similar average number of GVP ions was observed in the direct and modified NaOH + SDS methods, it is more reproducible with much smaller coefficient of variation (CV) in three experimental repeats in the direct method (0.02 vs. 0.27, respectively); and 4) gel blank—only a few peptide IDs from gel blank and no GVP identification at all. Gel blank serves as a control to see whether we introduce any contamination from handling the blank gel alone. Table 4 shows that the direct method is not only a more sensitive, but also a more reproducible method when compared to the modified NaOH + SDS method.

**Table 4:** Examination of reproducibility for the direct method and modified NaOH + SDS method* from a representative gel fraction (F6).

Methods (one 5 cm hair, Asian)	Yield (µg)	Main + Hair Spectral Library				
		Hair Proteome		Cuticular Keratins		GVP ions
		Proteins	Peptides	Proteins	Peptides	
Direct_R1	10.32	114	1427	14	593	43
Direct_R2	13.25	135	1617	15	620	44
Direct_R3	10.94	132	1725	14	618	45
NaOH + SDS_R1	3.36	101	1267	14	509	29
NaOH + SDS_R2	2.11	93	1178	14	497	51
NaOH + SDS_R3	3.32	83	1137	15	520	45
Blank Gel	0.04	6	17	2	7	0

* The result was obtained from fraction 6, a representative gel fraction. Three experimental repeats: R1, R2, and R3.

Estimation of the digestion yield: The gel‐based method we chose for analysis unfortunately did not allow us to use a conventional Bradford colorimetric assay (BCA) to measure protein concentration. Instead, yields of digested peptides using the Pierce method mentioned above served a similar, albeit less direct purpose. Based on a measured 5 cm hair mass of 100 µg (10 5 cm lengths were found to weigh 1.0 mg), we found that at the incubation time of 5, 10, 15, 30, 60, and 90 min, corresponding total yields of peptides to be 16%, 27%, 37%, 75%, 66%, and 51%. The maximum of 75% at 30 min was selected as optimal (see above). For comparison, a yield of 47% was reported for an in‐solution method 8 using BCA after precipitating extracted proteins.

### Examination of Artifacts Among Three Methods

In most proteomics experiments, a large fraction of ions sampled are not identified. This not only reduces the efficiency of the experiment but also has potential to generate false‐positive results. Moreover, the identity of the unidentified ions may aid in understanding and optimizing the experiment and provide a measure of quality control.

**Figure 7:**
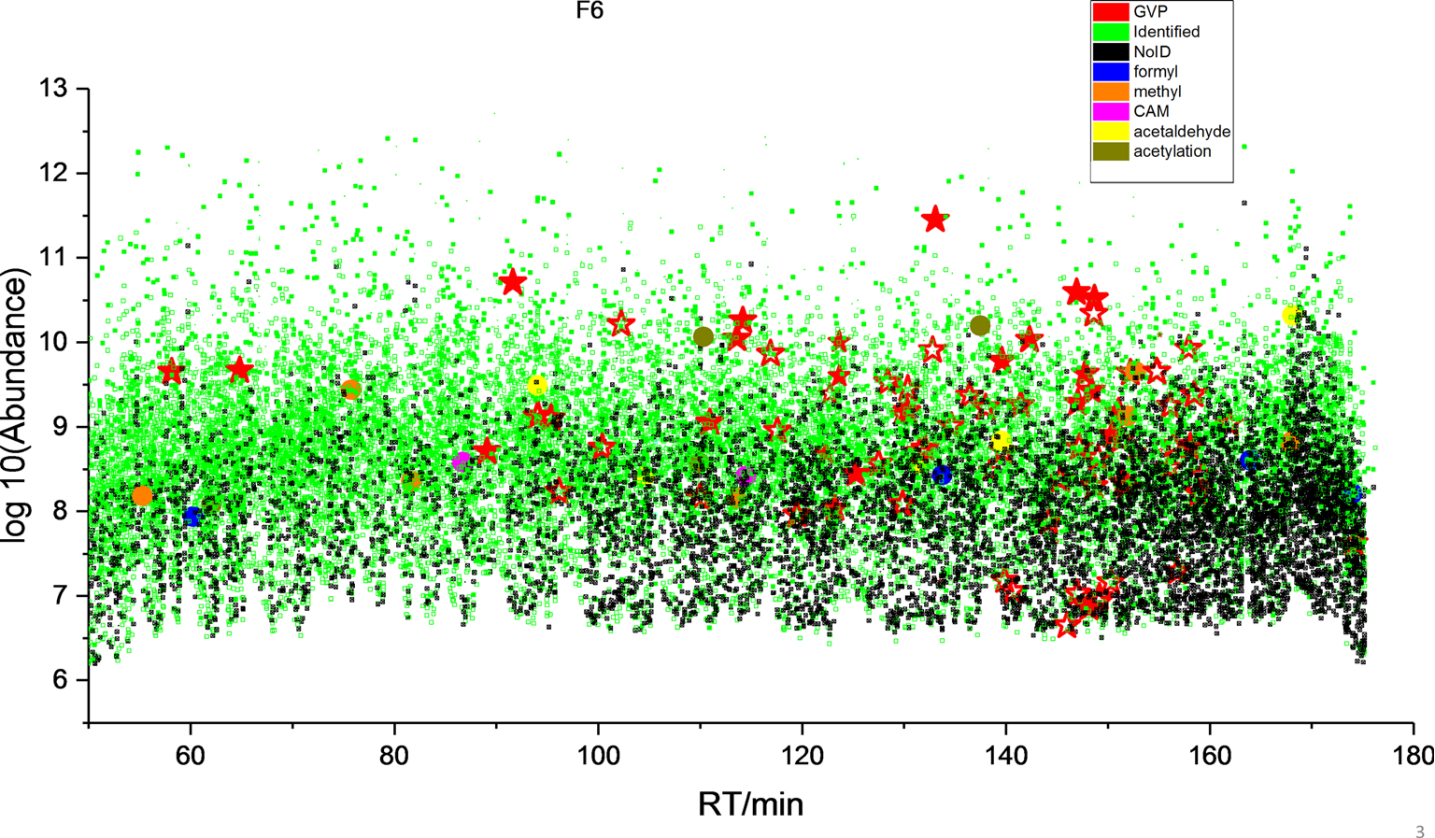
Classification of ions by the hybrid search. IonPlot shows the classification of GVP, identified, and not identified (NoID) ions, as well as several modifications: formylation (formyl), methylation (methyl), alkylation (CAM), acetaldehyde, and acetylation that present in fraction 6 (F6), a representative gel fraction from a protein gel separating proteins derived from a 5‐cm‐long hair shaft of this Asian donor by the direct method. Solid: identified by regular library search; hollowed: identified by hybrid library search. x‐axis: retention time (RT) in minute (min), y‐axis: abundance in log 10 scale.

In the present experiment, almost 90% of ions are not directly identified as tryptic peptides using conventional library searching. Using our recently developed hybrid search 15, as shown in Table S2, 11% can be identified as expected tryptic peptides, while about 75% can be identified via hybrid identification. These hybrid identifications find peptides that are chemically modified forms of conventional tryptic peptides. The reason we would like to examine experimentally introduced artifacts is because we must be aware of artifactual modifications that may masquerade as a GVP and therefore generate false‐positive identifications, the larger the number of spurious modifications the greater the chance that one will accidentally overlap a possible GVP. Proteomics cannot distinguish biological versus artifact origins of identified peptides. For example, a methylation at or near a serine might be interpreted as a serine‐to‐threonine GVP. IonPlot in Figure 7 shows the classification of ions (GVP, identified, and not identified ions from F6 of the direct method) by the hybrid search including a list of several interesting modifications that we would like to discuss more in this section. These analyses also show the nature and extent of certain spurious chemical processes that add to sample complexity and, in effect, diminish the sensitivity and overall quality of the experiment.

Since this issue is important for every sample preparation method regarding GVP detection, below we examine the artifacts among the three methods: our direct method, modified NaOH + SDS method, and cleavable surfactant method.

**Figure 8:**
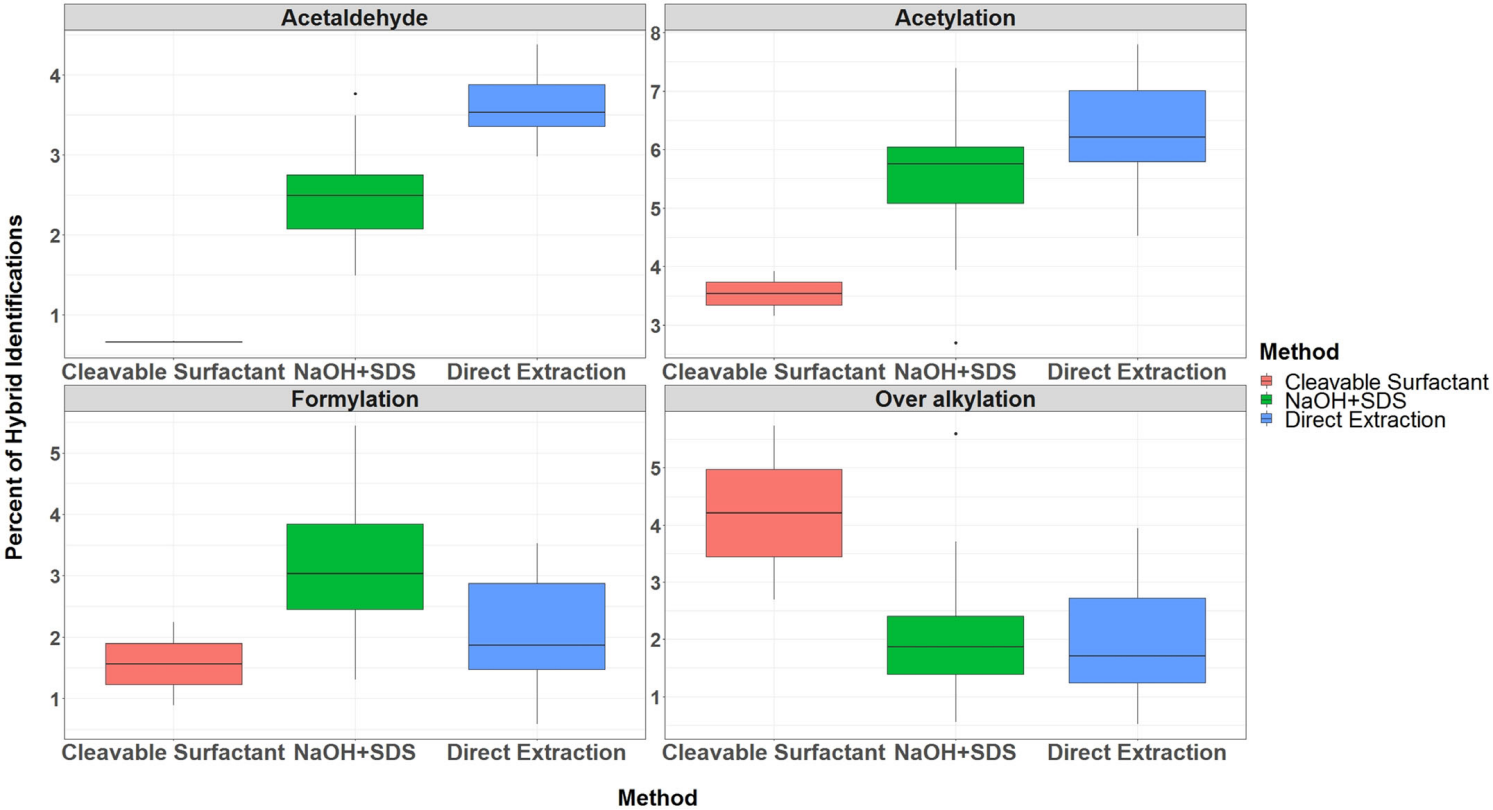
Comparison of the artifacts in the three methods. Comparison of experimentally introduced artifactual modifications among three methods using our recently developed hybrid search: cleavable surfactant method (red), modified NaOH + SDS method (green), and the direct method (blue). The compared experimentally introduced artifactual modifications chosen as examples are as follows: acetaldehyde (upper left), acetylation (upper right), formylation (lower left), and over alkylation (lower right).

Table 5 compares the twenty most frequently identified DeltaMass values in three methods 15. For more information, Appendix S4 shows the histograms of all DeltaMass values obtained from hybrid search identifications in each method to give a broad view of the distribution of all DeltaMass values. From the top 20 DeltaMass values listed in Table 5, we now further discuss four types of experimentally introduced artifactual modifications (Fig. 8).

**Table 5:** The twenty most frequently identified DeltaMass values obtained from hybrid search identifications in the three methods.

DeltaMass	Theoretical Value of DeltaMass	Proposed Modification	Percent of Hybrid Identifications		
			Direct (Median)	NaOH + SDS (Median)	Cleavable Surfactant (Median)
1.001	1.00335483	1‐C13	17.30	17.76	19.34
2.007	2.00670966	2‐C13	6.73	8.82	6.71
42.013	42.010565	Acetyl	6.25	5.75	3.54
26.017	26.015650	Acetaldehyde	3.52	2.49	0.66
3.009	3.01006449	3‐C13	3.59	4.96	3.55
27.999	27.994915	Formyl	1.87	3.03	1.57
14.018	14.015650	Methyl	3.08	2.60	1.12
‐1.011	‐1.00335483	‐1‐C13	2.31	3.05	
‐17.023	‐17.026549	‐NH3	1.62	1.51	2.38
70.007	70.005480	Formyl + Acetyl	0.89	1.28	
4.009	4.01341932	4‐C13	1.78	2.44	2.02
12.002	12.000000	Formaldehyde Adduct	1.45	1.20	
43.014	43.005814	Carbamyl/Acetyl + 1‐C13	1.48	1.07	0.70
‐18.008	‐18.010565	Dehydration/Glu → pyro‐Glu	1.34	1.35	2.01
‐2.013	‐2.00670966	‐2‐C13	1.36	1.58	1.43
23.986	23.98865266	Sodiated + 2C‐13	1.17		
57.023	57.021464	CAM	1.78	1.87	4.21
15.997	15.994915	Oxidation	1.08	1.28	
120.028	120.024500	Desulfurization + CAM + DTT	0.95		
58.010	58.005480	Deamidation + CAM	1.06	0.89	3.33
‐91.009	‐91.009185	Cys(CAM)→Dehydroalanine		0.82	
‐16.019	‐16.0231942	1C‐13 + ‐NH3		0.76	0.93
‐0.983	‐0.984016	Amidation			3.44
5.014	5.01677415	5‐C13			0.69
160.041	160.030654	Add‐Cys + CAM			1.25
31.995	31.989829	Dioxidation			1.78
152.003	151.996571	+DTT			0.86

**Figure 9:**
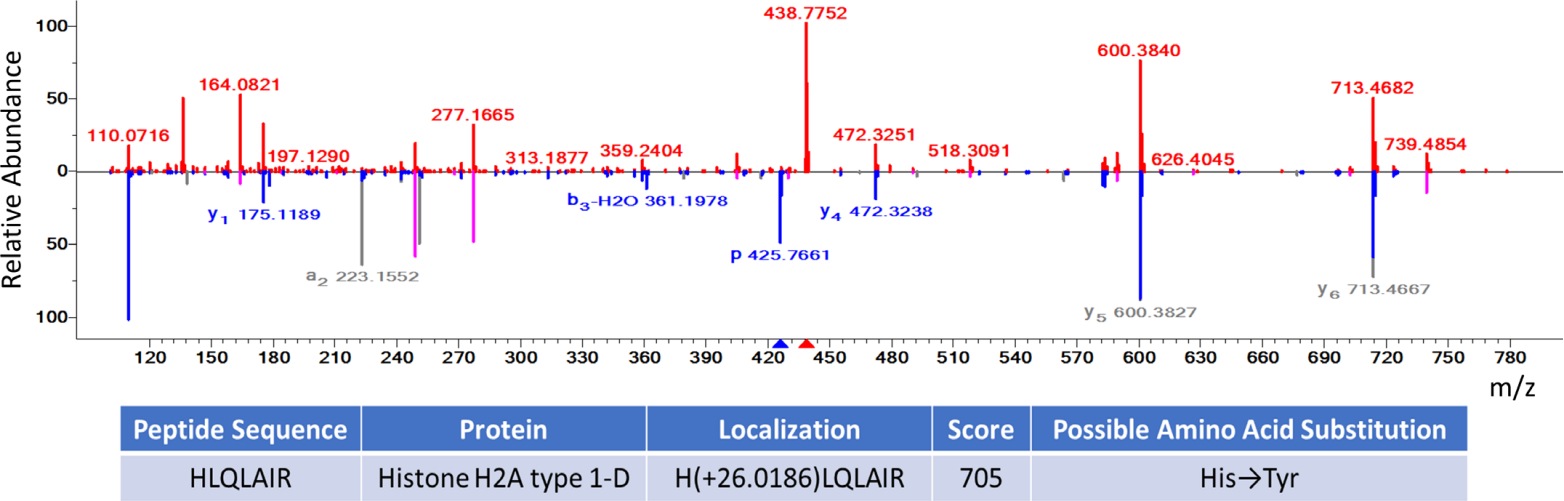
An example of a modification at peptide N‐terminus mistaken as a GVP. Spectral match of a hair‐derived peptide to the peptide sequence HLQLAIR (Charge = 2, Mods = 0, Spectral Match Score = 705) with a DeltaMass of 26.0186 Da, which is likely due to acetaldehyde (26.01565 Da) but may be incorrectly identified as His (H) →Tyr (Y) (26.004417 Da).

Acetaldehyde adduction: We compared the occurrence of an acetaldehyde adduct across the three methods. Figure 8 shows that this artifactual modification is more frequently identified in the direct and modified NaOH + SDS methods due to the presence of ethanol in the SimplyBlue SafeStain that we used to stain the protein gels. We here included an example in Figure 9 to show our main concern—a modification at peptide’s N‐terminus could be mistaken as a potential GVP: The DeltaMass value from the hybrid search for this hybrid identification is 26.0186 Da, within the mass tolerance range, which is likely due to acetaldehyde (26.01565 Da) but may be incorrectly identified as His (H) →Tyr (Y) (26.004417 Da) since His (H) is involved in the identification at the first amino acid in this peptide ion. Without the hybrid search, or without being aware of what type of artifactual modification exists, such a misidentification will occur.

Acetylation: While acetylation at Lys (K) and the protein amino terminus is biological modifications, artifactual acetylation at the peptide N‐terminus can be introduced during sample preparation. Although the source of acetic acid is not believed to have been introduced through sample preparation, this artifactual modification was identified more frequently in the direct and modified NaOH + SDS methods.

Formylation: Formylation is less dissimilar across all three methods than that of the previously described two modifications. This is expected as formic acid is required in all three sample preparations.

Alkylation: Alkylation (CAM) is significantly greater in the cleavable surfactant method compared with the direct and modified NaOH + SDS methods. This is consistent with the fact that iodoacetamide concentration we used in sample preparation of cleavable surfactant method is much higher than in the direct and modified NaOH + SDS methods.

Table 5 and Appendix S4 show that, overall, the results of the three methods have similar degrees of experimentally introduced modifications. It seems likely that the artefactual modifications are a result of the inherent difficulty of digestion such an insoluble and cross‐linked material as hair.

Regarding GVP panel analysis, we find consistent results in regular and hybrid searches. Hybrid searching usually reports more GVP ions with many kinds of unexpected modifications but seems not gaining additional known GVP site detection. Verified GVP detection by the hybrid search (not only seeing the version that included in the library but also seeing the versions with some unexpected modifications) increases the confidence of GVP panel analysis.

### Identification of Hair Proteome and Cuticular Keratins from as Little as 1‐cm‐long Human Hair Shaft by Direct Extraction Method

**Figure 10:**
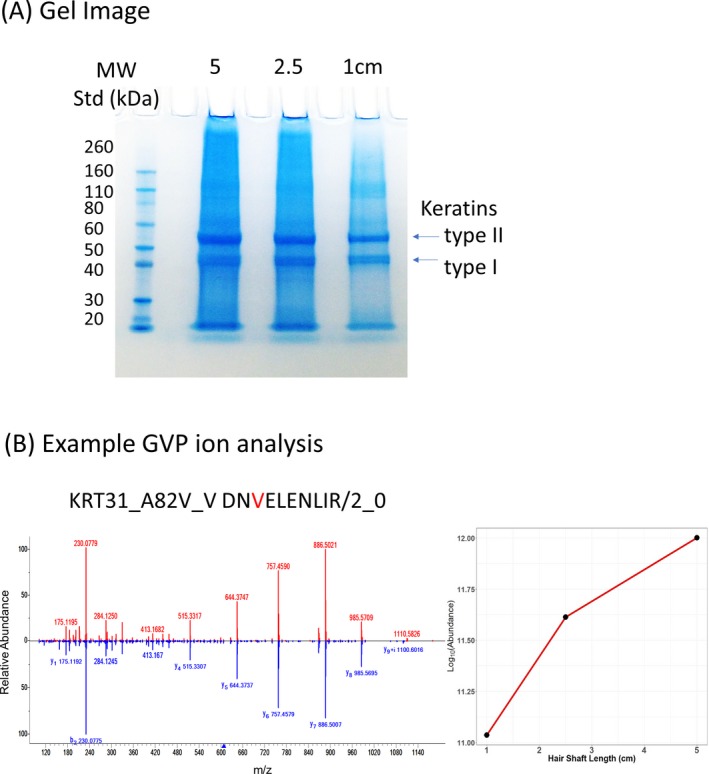
Comparison of hair length variation. Comparison of hair length variation. (A) This gel image shows the separation of hair proteins from 5‐, 2.5‐, and 1‐cm‐long hair shaft samples from the same individual donor. A MW standard was loaded in the first lane. Bands for type I and type II hair cuticular keratins were labeled. (B) Spectral match (MF = 921) of an example GVP ion (KRT31_A82V_V: DN[V]ELENLIR/2_0 HCD = 30eV) is on the left. The spectrum shown in red is the query spectrum, and the spectrum shown in blue is the reference library spectrum for this GVP ion. On the right is a plot that shows the abundance of this example GVP ion in the 1‐, 2.5‐, and 5‐cm hair shaft samples is approximately linear. Note the y‐axis is the log of the abundance value, plotted on a linear scale.

So far, the data we presented in this manuscript used 5‐cm‐long hair shafts as the starting material. While we learned about the sensitivity of the direct method with the serial dilution study, we also wanted to check results using smaller lengths of hair. As the dilution series was a projection for low amounts based on similar extraction efficiencies for smaller lengths, one may expect further losses due to possible inefficiencies in digesting small lengths of hair. For this purpose, we undertook a series of studies where hair shaft varied from 5, 2.5, and 1 cm long. Figure 10A shows the separation of hair proteins by SDS‐PAGE for three different hair lengths, and Table 6 lists the total number of hair proteins and peptides identified as well as those that are specific for hair cuticular keratins and GVP ions. Figure 10B shows the analysis of an example GVP ion whose abundance is almost linear in 5‐, 2.5‐, and 1‐cm hair shaft samples to demonstrate the abundance is proportional to length. These results show that as little as 1‐cm‐long hair shaft sample can be analyzed by this direct method. There is no reason to believe it would not work effectively for even smaller amounts of hair, suggesting that even forensic‐relevant trace quantities of hair would be suitable for this analytical method.

**Table 6:** Reduction in starting material to 1‐cm‐long hair shaft by the direct method.*

Hair Length (cm)	Main + Hair Spectral Library				
	Hair Proteome		Cuticular Keratins		GVP ions
	Proteins	Peptides	Proteins	Peptides	
5	135	1617	15	620	44
2.5	86	1203	14	563	40
1	78	1149	14	486	39

* The result was obtained from fraction 6, a representative gel fraction.

### Examination of the Direct Method in Another Donor

To ensure that these results were not unique to one donor, we applied the direct method to another randomly selected donor’s hair shaft samples obtained from BioreclamationIVT (LOT# BRH1363733, 5 g of hair shafts from a Caucasian male, 23 years old). Table 7 lists the total number of hair proteins and peptides identified as well as those from hair cuticular keratins and GVP ions. These results demonstrate that the direct method works equally well for another donor’s hair samples. The overall protein gel images, the peptide yields from in‐gel digestions, the hair keratins and their peptide identifications, and the number of found GVP ions are similar. Most of the high‐abundance GVPs in this Caucasian donor overlap with previously described Asian donor in the GVP panel analysis. This manuscript is focused on the protein and peptide extraction from single hair shaft, that is the reason why we use hair samples from the same Asian donor for the development of protein extraction method. We believe our direct method would work effectively for hair samples from any individual donor. These studies did not consider donors who heated or chemically treated their hair—this would be a useful topic for future research. The focus of this paper was only analytical methods and detailed proteomic analysis. Variations with hair origin will be the topic of future studies using the methods described here.

**Table 7:** Comparison of protein and peptide identification from a 5‐cm‐long hair shaft from Asian and Caucasian male donor by the direct method.*

Donor	Yield (µg)	Main + Hair Spectral Library				
		Hair Proteome		Cuticular Keratins		GVP Ions
		Proteins	Peptides	Proteins	Peptides	
Asian	13.25	135	1617	15	620	44
Caucasian	8.48	92	1177	14	581	45

* The result was obtained from fraction 6, a representative gel fraction.

## Summary and Conclusions

In summary, we have shown that the direct extraction method is a sensitive, reliable, and relatively convenient method based on the depth of coverage of the human hair proteome and cuticular keratins: (i) It is a relatively sensitive method: It works for a hair shaft as short as 1 cm; (ii) it is a relatively reliable method: It generates more consistent results in protein/peptide identification and GVP detection; and (iii) it is a relatively convenient method: It is simple to carry out since there is only one step in protein extraction from hair, although to assure maximum GVP identification, it does require multiple LC‐MS/MS runs.

Using our recently developed “hybrid” spectral library search method, we have found that a very large fraction of the peptide spectra acquired were not simple tryptic peptides derived from known proteins. A conventional library search can identify only 11% of the peptides, while the hybrid search identifies 75%, including any previously unidentified GVPs (as our future work). We have also shown that the hybrid search could be used to identify potential sources of false positives due to the presence of artifactual modifications that are experimentally introduced. Modifications that could be mistaken as a GVP should be the primary concern, and a separated examination of artefactual modifications is needed. In difficult cases, a more careful manual checking of GVP spectra may also be needed.

Although we recommend the direct method because of several advantages we described earlier, we also realize different methods may be most suitable for different GVP panel analysis. Each method will have its own strength and weakness. Unless we combine the results from all three tested methods, no single method covered all the identified published GVP sites in this study. This is largely because of the nature of the hair samples—heavy cross‐linking makes hair mechanically strong and stable, but also very resistant to sample processing.

We have also shown that a GVP analysis can effectively done using a peptide spectral library containing all identifiable peptides derived from human hair samples. With this paper, we provide a library containing all identified hair‐derived peptides 13. Future expansion of this library can include all known GVPs as well as all identifiable peptides derived from human hair. Further, it may be combined with the NIST‐developed label‐free HCD main peptide library (peptide.nist.gov) 12 to provide another layer of sensitivity and confidence for hair peptide identification and GVP detection.

## Supporting information


**Appendix S1.** Outline of protein extraction workflows for direct method and modified NaOH + SDS method.

**Appendix S2.** Comparison of sequences coverage in amino acids of 15 type I and type II hair cuticular keratins by library and Sequest searching.

**Appendix S3.** GVP panel analyses in all ten fractions by the direct method.

**Appendix S4.** Histograms of the distribution of all DeltaMass values in three methods.

**Table S1.** Example of a big protein and a small protein amount change in ten gel fractions by the direct method.

**Table S2.** Percentages of hybrid IDs in all ten gel fractions by the direct method.

## References

[1] Parker GJ , Leppert T , Anex DS , Hilmer JK , Matsunami N , Baird L , et al. Demonstration of protein‐based human identification using the hair shaft proteome. PLoS ONE 2016;11(9):e0160653. 27603779 10.1371/journal.pone.0160653 PMC5014411

[2] Mason KE , Paul PH , Chu F , Anex DS , Hart BR . Development of a protein‐based human identification capability from a single hair. J Forensic Sci 2019;64(4):1152–9. 30735575 10.1111/1556-4029.13995

[3] Carlson TL , Moini M , Eckenrode BA , Allred BM , Donfack J . Protein extraction from human anagen head hairs 1‐millimeter or less in total length. Biotechniques 2018;64(4):170–6. 29661011 10.2144/btn-2018-2004

[4] Bengtsson CF , Olsen ME , Brandt LØ , Bertelsen MF , Willerslev E , Tobin DJDNA , et al. from keratinous tissue. Part I: hair and nail. Ann Anat 2012;194(1):17–25. 21530205 10.1016/j.aanat.2011.03.013

[5] Langbein L , Rogers MA , Winter H , Praetzel S , Beckhaus U , Rackwitz HR , et al. The catalog of human hair keratins. I. Expression of the nine type I members in the hair follicle. J Biol Chem 1999;274(28):19874–84. 10391933 10.1074/jbc.274.28.19874

[6] Langbein L , Rogers MA , Winter H , Praetzel S , Schweizer J . The catalog of human hair keratins. II. Expression of the six type II members in the hair follicle and the combined catalog of human type I and II keratins. J Biol Chem 2001;276(37):35123–32. 11445569 10.1074/jbc.M103305200

[7] Lee YJ , Rice RH , Lee YM . Proteome analysis of human hair shaft: from protein identification to posttranslational modification. Mol Cell Proteomics 2006;5(5):789–800. 16446289 10.1074/mcp.M500278-MCP200

[8] Wong SY , Lee CC , Ashrafzadeh A , Junit SM , Abrahim N , Hashim OH . A high‐yield two‐hour protocol for extraction of human hair shaft proteins. PLoS ONE 2016;11(10):e0164993. 27741315 10.1371/journal.pone.0164993 PMC5065217

[9] Adav SS , Subbaiaih RS , Kerk SK , Lee AY , Lai HY , Ng KW , et al. Studies on the proteome of human hair – identification of histones and deamidated keratins. Sci Rep 2018;8(1):1599. 29371649 10.1038/s41598-018-20041-9 PMC5785504

[10] Jimenez CR , Huang L , Qiu Y , Burlingame AL . In‐gel digestion of proteins for MALDI‐MS fingerprint mapping. Curr Protoc Protein Sci 2001;14(1):16.4.1–5. 10.1002/0471140864.ps1604s14 18429131

[11] Rudnick PA , Markey SP , Roth J , Mirokhin Y , Yan X , Tchekhovskoi DV , et al. A description of the clinical proteomic tumor analysis consortium (CPTAC) common data analysis pipeline. J Proteome Res 2016;15(3):1023–32. 26860878 10.1021/acs.jproteome.5b01091 PMC5117628

[12] The NIST main libraries of peptide tandem mass spectra. https://chemdata.nist.gov/dokuwiki/doku.php?xml:id=peptidew:lib:humanhcd20160503 (accessed October 14, 2019).

[13] The NIST hair libraries of peptide tandem mass spectra. https://chemdata.nist.gov/dokuwiki/doku.php?xml:id=peptidew:lib:human_hair_selected_with_gvps_passed (accessed October 14, 2019).

[14] Zhang Z , Burke M , Mirokhin YA , Tchekhovskoi DV , Markey SP , Yu W , et al. Reverse and random decoy methods for false discovery rate estimation in high mass accuracy peptide spectral library searches. J Proteome Res 2018;17(2):846–57. 29281288 10.1021/acs.jproteome.7b00614

[15] Burke MC , Mirokhin YA , Tchekhovskoi DV , Markey SP , Heidbrink Thompson J , Larkin C , et al. The hybrid search: a mass spectral library search method for discovery of modifications in proteomics. J Proteome Res 2017;16(5):1924–35. 28367633 10.1021/acs.jproteome.6b00988

[16] Eng JK , McCormack AL , Yates JR . An approach to correlate tandem mass spectral data of peptides with amino acid sequences in a protein database. J Am Soc Mass Spectrom 1994;5(11):976–89. 24226387 10.1016/1044-0305(94)80016-2

[17] Abbatiello SE , Schilling B , Mani DR , Zimmerman LJ , Hall SC , MacLean B , et al. Large‐scale interlaboratory study to develop, analytically validate and apply highly multiplexed, quantitative peptide assays to measure cancer‐relevant proteins in plasma. Mol Cell Proteomics 2015;14(9):2357–74. 25693799 10.1074/mcp.M114.047050 PMC4563721

[18] Speicher K , Kolbas O , Harper S , Speicher D . Systematic analysis of peptide recoveries from in‐gel digestions for protein identifications in proteome studies. J Biomol Tech 2000;11(2):74–86. 19499040 PMC2291619

[19] Lowenthal MS , Liang Y , Phinney KW , Stein SE . Quantitative bottom‐up proteomics depends on digestion conditions. Anal Chem 2014;86(1):551–8. 24294946 10.1021/ac4027274

