# Supplementary material for: Sensitive Method for the Confident Identification of Genetically Variant Peptides in Human Hair Keratin
Source: J Forensic Sci. 2019 Oct 31;65(2):406–20. doi: 10.1111/1556-4029.14229 (PMC7064992; doi:10.1111/1556-4029.14229)
Supplement: Supplementary file 1 — Appendix S1. Outline of protein extraction workflows for direct method and modified NaOH + SDS method. [file JFO-65-406-s001.docx]

SUPPLEMENTARY DOCUMENT S1—*Outline of protein extraction work flows for direct method and modified NaOH+SDS method.*

1. Direct Method


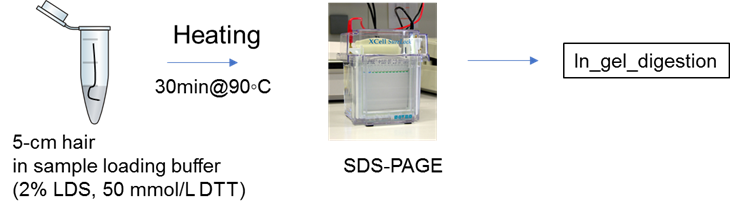


1. Modified NaOH+SDS Method


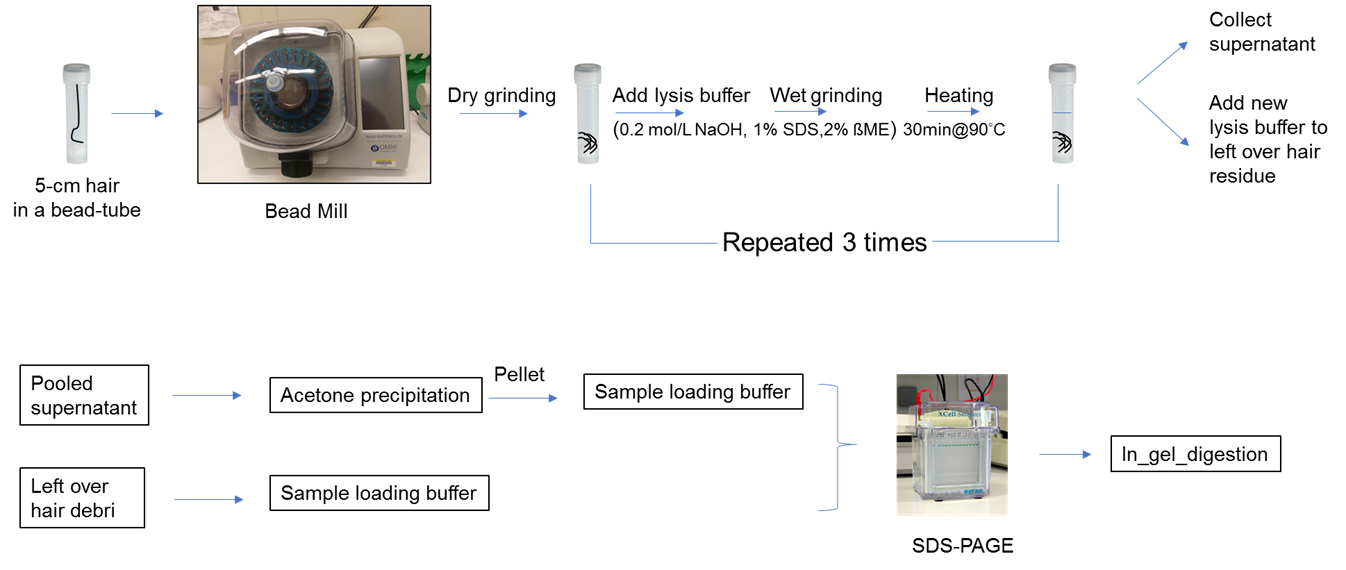


Workflows of the direct method and modified NaOH+SDS method are illustrated.
