# Supplementary material for: Sensitive Method for the Confident Identification of Genetically Variant Peptides in Human Hair Keratin
Source: J Forensic Sci. 2019 Oct 31;65(2):406–20. doi: 10.1111/1556-4029.14229 (PMC7064992; doi:10.1111/1556-4029.14229)
Supplement: Supplementary file 2 — Appendix S2. Comparison of sequences coverage in amino acids of 15 type I and type II hair cuticular keratins by library and Sequest searching. [file JFO-65-406-s002.docx]

SUPPLEMENTARY DOCUMENT S2—*Comparison of sequences coverage in amino acids of 15 type I and type II hair cuticular keratins by library and sequest searching. Amino acid sequence highlighted in green indicates peptide identified with high confidence (FDR at 1% level) by Sequest and library searching; in yellow indicates peptide identified with high confidence* ***by library searching only****. This sheet is sorted by type I cuticular keratins (from KRT31 to KRT38) and type II cuticular keratins (from KRT81 to KRT86). The coverage analyses were combined from all ten gel fractions.*

**GN=KRT31: Keratin, type I cuticular Ha1 OS=Homo sapiens**

**From Library (100%) and Sequest (97.6%):**

M**PYNFCLPSL SCRTSCSSRP CVPPSCHSCT LPGACNIPAN VSNCNWFCEG SFNGSEKETM QFLNDR**LASY

LEKVR**QLERD NAELENLIRE RSQQQEPLLC PSYQSYFKTI EELQQKILCT KSENARLVVQ IDNAKLAADD**

**FRTKYQTELS LRQLVESDIN GLRRILDELT LCKSDLEAQV ESLKEELLCL KSNHEQEVNT LRCQLGDRLN**

**VEVDAAPTVD LNRVLNETRS QYEALVETNR REVEQWFTTQ TEELNKQVVS SSEQLQSYQA EIIELRRTVN**

**ALEIELQAQH NLRDSLENTL TESEARYSSQ LSQVQSLITN VESQLAEIRS DLERQNQEYQ VLLDVRARLE**

**CEINTYRSLL ESEDCNLPSN PCATTNACSK PIGPCLSNPC TSCVPPAPCT PCAPRPRCGP CNSFVR**

**GN=KRT32: Keratin, type I cuticular Ha2 OS=Homo sapiens**

**From Library (54.2%) and Sequest (49.6%):**

MTSSCCVTNN LQASLKSCPR PASVCSSGVN CRPELCLGYV CQPMACLPSV CLPTTFRPAS CLSKTYLSSS

CQAASGISGS MGPGSWYSEG AFNGNEK**ETM QFLNDR**LASY LTRVR**QLEQE NAELESRIQE ASHSQVLTMT**

**PDYQSHFRTI EELQQKILCT K**AENAR**MVVN IDNAKLAADD FRAKYEAELA MRQLVEADIN GLRRILDDLT**

**LCKADLEAQV ESLKEELMCL KKNHEEEVGS LR**CQLGDR**LN IEVDAAPPVD LTR**VLEEMRC QYEAMVEANR

RDVEEWFNMQ MEELNQQVAT SSEQLQNYQS DIIDLRR**TVN TLEIELQAQH SLRDSLENTL TESEARYSSQ**

**LAQMQCMITN VEAQLAEIR**A DLE**RQNQEYQ VLLDVRARLE GEINTYR**SLL ENEDCKLPCN PCSTPSCTTC

VPSPCVPRTV CVPRTVGMPC SPCPQGRY

**GN=KRT33A: Keratin, type I cuticular Ha3-I OS=Homo sapiens**

**From Library (97.0%) and Sequest (93.3%):**

M**SYSCGLPSL SCRTSCSSRP CVPPSCHGCT LPGACNIPAN VSNCNWFCEG SFNGSEKETM QFLNDR**LASY

LEKVR**QLERD NAELENLIRE RSQQQEPLVC ASYQSYFKTI EELQQKILCS KSENARLVVQ IDNAKLASDD**

**FRTKYETELS LRQLVESDIN GLRRILDELT LCRSDLEAQV ESLKEELLCL KQNHEQEVNT LR**CQLGDR**LN**

**VEVDAAPTVD LNQVLNETRS QYEALVETNR REVEQWFATQ TEELNKQVVS SSEQLQSYQA EIIELRRTVN**

**ALEIELQAQH NLRDSLENTL TESEARYSSQ LSQVQRLITN VESQLAEIRS DLERQNQEYQ VLLDVRARLE**

**CEINTYRSLL ESEDCKLPSN PCATTNACDK STGPCISNPC GLR**ARCGPCN TFGY

**GN=KRT33B: Keratin, type I cuticular Ha3-II OS=Homo sapiens**

**From Library (97.0%) and Sequest (93.6%):**

M**PYNFCLPSL SCRTSCSSRP CVPPSCHGYT LPGACNIPAN VSNCNWFCEG SFNGSEKETM QFLNDR**LASY

LEKVR**QLERD NAELENLIRE RSQQQEPLLC PSYQSYFKTI EELQQKILCS KSENARLVVQ IDNAKLAADD**

**FRTKYQTEQS LRQLVESDIN SLRRILDELT LCRSDLEAQM ESLKEELLSL KQNHEQEVNT LR**CQLGD**RLN**

**VEVDAAPAVD LNQVLNETRN QYEALVETNR REVEQWFATQ TEELNKQVVS SSEQLQSYQA EIIELRRTVN**

**ALEIELQAQH NLRYSLENTL TESEARYSSQ LSQVQSLITN VESQLAEIRS DLERQNQEYQ VLLDVRARLE**

**CEINTYRSLL ESEDCKLPSN PCATTNACEK PIGSCVTNPC GPR**SRCGPCN TFGY

**GN=KRT34:** **Keratin, type I cuticular Ha4 OS=Homo sapiens**

**From Library (86.0%) and Sequest (83.9%):**

MLYAKPPPTI NGIKGLQRKE RLKPAHIHLQ QLTCFSITCS STM**SYSCCLP SLGCRTSCSS RPCVPPSCHG**

**YTLPGACNIP ANVSNCNWFC EGSFNGSEKE TMQFLNDR**LA SYLEKVR**QLE RDNAELEKLI QERSQQQEPL**

**LCPSYQSYFK TIEELQQKIL CAKAENARLV VNIDNAKLAS DDFRSKYQTE QSLRLLVESD INSIRRILDE**

**LTLCKSDLES QVESLREELI CLKKNHEEEV NTLRSQLGDR LNVEVDTAPT VDLNQVLNET R**SQYEALVEI

N**RREVEQWFA TQTEELNKQV VSSSEQLQSC QAEIIELRRT VNALEIELQA QHNLRDSLEN TLTESEAHYS**

**SQLSQVQSLI TNVESQLAEI R**CDLE**RQNQE YQVLLDVRAR LECEINTYRS LLESEDCKLP CNPCATTNAS**

**GNSCGPCGTS QK**GCCN

**GN=KRT35:** **Keratin, type I cuticular Ha5 OS=Homo sapiens**

**From Library (91.0%) and Sequest (86.4%):**

MASKCLK**AGF SSGSLKSPGG ASGGSTRVSA MYSSSSCKLP SLSPVARSFS ACSVGLGR**SS YR**ATSCLPAL**

**CLPAGGFATS YSGGGGWFGE GILTGNEKET MQSLNDR**LAG YLEKVR**QLEQ ENASLESRIR EWCEQQVPYM**

**CPDYQSYFR**T IEELQKKTLC SKAENAR**LVV EIDNAKLAAD DFRTKYETEV SLRQLVESDI NGLRRILDDL**

**TLCKSDLEAQ VESLKEELLC LKKNHEEEVN SLR**CQLGDR**L NVEVDAAPPV DLNR**VLEEMR **CQYETLVENN**

RR**DAEDWLDT QSEELNQQVV SSSEQLQSCQ AEIIELRRTV NALEIELQAQ HSMRDALEST LAETEARYSS**

**QLAQMQCMIT NVEAQLAEIR** ADLE**RQNQEY QVLLDVRARL ECEINTYRGL LESEDSKLPC NPCAPDYSPS**

K**SCLPCLPAA SCGPSAAR**TN CSPR**PICVPC PGGR**F

**GN=KRT36:** **Keratin, type I cuticular Ha6 OS=Homo sapiens**

**From Library (60.8%) and Sequest (49.3%):**

MATQTCTPTF STGSIKGLCG TAGGISRVSS IRSVGSCR**VP SLAGAAGYIS SAR**SGLSGLG SCLPGSYLSS

ECHTSGFVGS GGWF**CEGSFN GSEKETMQFL NDR**LANYLEK VRQLERENAE LESRIQEWYE FQIPYICPDY

QSYFKTIEDF QQKILLTKSE NARLVL**QIDN AKLAADDFRT KYETELSLRQ LVEADINGLR** **RILDELTLCK**

**ADLEAQVESL KEELMCLK**K**N HEEEVSVLR**C QLGDR**LNVEV DAAPPVDLNK** ILEDMRCQYE ALVENNRRDV

EAWFNTQTEE LNQQVVSSSE QLQCCQTEII ELR**RTVNALE IELQAQHSMR** **NSLESTLAET EARYSSQLAQ**

**MQCLISNVEA QLSEIR**CDLE R**QNQEYQVLL DVK**AR**LEGEI ATYRHLLEGE DCK**LPPQPCA TACKPVIR**VP**

**SVPPVPCVPS VPCTPAPQVG TQIR**TITEEI RDGKVISSRE HVQSRPL

**GN=KRT37:** **Keratin, type I cuticular Ha7 OS=Homo sapiens**

**From Library (43.0%) and Sequest (34.7%):**

MTSFYSTSSC PLGCTMAPGA RNVFVSPIDV GCQPVAEANA ASM**CLLANVA HANR**VRVGST PLGRPSLCLP

PTSHTACPLP GTCHIPGNIG ICGAYGKNTL NGHEKETMKF LNDRLANYLE KVR**QLEQENA ELETTLLER**S

KCHESTVCPD YQSY**FRTIEE LQQKILCSK**A ENAR**LIVQID NAKLAADDFR** IKLESERSLH QLVEADKCGT

QKLLDDATLA K**ADLEAQQES LKEEQLSLK**S NHEQEVKILR SQLGEKFR**IE LDIEPTIDLN R**VLGEMRAQY

EAMVETNHQD VEQWFQAQSE GISLQAMSCS EELQCCQSEI LELR**CTVNAL EVER**QAQHTL K**DCLQNSLCE**

**AEDRYGTELA QMQSLISNLE EQLSEIR**ADL ER**QNQEYQVL LDVK**AR**LENE IATYR**NLLES EDCKLPCNPC

STPASCTSCP SCGPVTGGSP SGHGASMGR

**GN=KRT38: Keratin, type I cuticular Ha8 OS=Homo sapiens**

**From Library (61.2%) and Sequest (51.3%):**

MTSSYSSSSC PLGCTMAPGA R**NVSVSPIDI GCQPGAEANI APMCLLANVA HANR**VRVGST PLGRPSLCLP

PTCHTACPLP GTCHIPGNIG ICGAYGENTL NGHEK**ETMQF LNDR**LANYLE KVR**QLEQENA ELEATLLER**S

K**CHESTVCPD YQSYFHTIEE LQQKILCSK**A ENAR**LIVQID NAKLAADDFR** IKLESERSLR QLVEADKCGT

QKLLDDATLA K**ADLEAQQES LKEEQLSLK**S NHEQEVKILR SQLGEKLR**IE LDIEPTIDLN R**VLGEMR**AQY**

**EAMLETNR**QD VEQWFQAQSE GISLQDMSCS EELQCCQSEI LELR**CTVNAL EVER**QAQHTL K**DCLQNSLCE**

**AEDRFGTELA QMQSLISNVE EQLSEIR**ADL ER**QNQEYQVL LDVKTRLENE IATYR**NLLES EDCKLPCNPC

STSPSCVTAP CAPR**PSCGPC TTCGPTCGAS TTGSR**F

**GN=KRT81: Keratin, type II cuticular Hb1 OS=Homo sapiens**

**From Library (96.2%) and Sequest (91.9%):**

M**TCGSGFGGR** **AFSCISACGP RPGRCCITAA PYR**GISCY**RG LTGGFGSHSV CGGFR**AGSCG RSFGYR**SGGV**

**CGPSPPCITT VSVNESLLTP LNLEIDPNAQ CVKQEEKEQI KSLNSRFAAF IDKVRFLEQQ NKLLETKLQF**

**YQNRECCQSN LEPLFEGYIE TLRREAECVE ADSGRLASEL NHVQEVLEGY KKKYEEEVSL RATAENEFVA**

**LKKDVDCAYL RKSDLEANVE ALIQEIDFLR RLYEEEILIL QSHISDTSVV VKLDNSRDLN MDCIIAEIKA**

**QYDDIVTRSR AEAESWYRSK CEEMKATVIR** HGETLR**RTKE EINELNR**MIQ **RLTAEVENAK CQNSKLEAAV**

**AQSEQQGEAA LSDARCKLAE LEGALQKAKQ DMACLIREYQ EVMNSKLGLD IEIATYRRLL EGEEQRLCEG**

**IGAVNVCVSS SRGGVVCGDL CVSGSRPVTG SVCSAPCN**GN VAVSTGLCAP C**GQLNTTCGG GSCGVGSCGI**

**SSLGVGSCGS SCR**KC

**GN=KRT82: Keratin, type II cuticular Hb2 OS=Homo sapiens**

**From Library (63.4%) and Sequest (49.9%):**

MSYHSFQPGS RCGSQSFSSY SAVMPRMVTH YAVSKGPCRP GGGRGLRALG CLGSRSLCNV GFGRPRVASR

**CGGTLPGFGY RLGATCGPSA CITPVTINES LLVPLALEID PTVQR**VKRDE KEQIKCLNNR FASFINKVRF

LEQKNKLLET KWNFMQQQR**C CQTNIEPIFE GYISALR**R**QL DCVSGDRVRL ESELCSLQAA LEGYKK**K**YEE**

**ELSLRPCVEN EFVALKK**DVD TAFLMK**ADLE TNAEALVQEI DFLKSLYEEE ICLLQSQISE TSVIVK**MDNS

R**ELDVDGIIA EIK**AQYDDIA SR**SKAEAEAW YQCR**YEELR**V TAGNHCDNLR** NRKNEILEMN KLIQRLQQET

ENVKAQRCK**L EGAIAEAEQQ GEAALNDAK**C K**LAGLEEALQ KAKQDMACLL KEYQEVMNSK LGLDIEIATY**

**RRLLEGEEHR L**CEGIGPVNI SVSSSKGAFL YEPCGVSTPV LSTGVLRSNG GCSIVGTGEL YVPCEPQGLL

SCGSGRKSSM TLGAGGSSPS HKH

**GN=KRT83: Keratin, type II cuticular Hb3 OS=Homo sapiens**

**From Library (97.0%) and Sequest (87.2%):**

MTCGFNSIGC GFRPGNFSCV SACGPRPS**RC CITAAPYR**GI SCY**RGLTGGF GSHSVCGGFR** AGSCGRSFGY

R**SGGVCGPSP PCITTVSVNE SLLTPLNLEI DPNAQCVKQE EKEQIKSLNS RFAAFIDKVR FLEQQNKLLE**

**TKLQFYQNRE CCQSNLEPLF AGYIETLRRE AECVEADSGR LASELNHVQE VLEGYKKKYE EEVALRATAE**

**NEFVALKKDV DCAYLRKSDL EANVEALIQE IDFLRRLYEE EIRILQSHIS DTSVVVKLDN SRDLNMDCIV**

**AEIKAQYDDI ATRSRAEAES WYRSKCEEMK ATVIR**HGETL R**RTKEEINEL NR**MIQ**RLTAE VENAKCQNSK**

**LEAAVAQSEQ QGEAALSDAR CKLAELEGAL QKAKQDMACL IREYQEVMNS KLGLDIEIAT YRRLLEGEEQ**

**RLCEGVEAVN VCVSSSRGGV VCGDLCVSGS RPVTGSVCSA PCN**GNLVVST GL**CKPCGQLN TTCGGGSCGQ**

**GR**H

**GN=KRT84: Keratin, type II cuticular Hb4 OS=Homo sapiens**

**From Library (12.7%) and Sequest (11.2%):**

MSCRSYRVSS GHRVGNFSSC SAMTPQNLNR FRANSVSCWS GPGFRGLGSF GSRSVITFGS YSPRIAAVGS

RPIHCGVRFG AGCGMGFGDG RGVGLGPRAD SCVGLGFGAG SGIGYGFGGP GFGYRVGGVG VPAAPSITAV

TVNKSLLTPL NLEIDPNAQR VKKDEKEQIK TLNNK**FASFI DKVRFLEQQN KLLETK**WSFL QEQKCIRSNL

EPLFESYITN LRRQLEVLVS DQARLQAERN HLQDVLEGFK KKYEEEVVCR **ANAENEFVAL KK**DVDAAFMN

KSDLEANVDT LTQEIDFLKT LYMEEIQLLQ SHISETSVIV KMDNSRDLNL DGIIAEVKAQ YEEVARRSRA

DAEAWYQTKY EEMQVTAGQH CDNLRNIRNE INELTRLIQR LKAEIEHAKA QR**AKLEAAVA EAEQQGEATL**

**SDAK**CKLADL ECALQQAKQD MARQLCEYQE LMNAK**LGLDI EIATYRR**LLE GEESRLCEGV GPVNISVSSS

RGGLVCGPEP LVAGSTLSRG GVTFSGSSSV CATSGVLASC GPSLGGARVA PATGDLLSTG TRSGSMLISE

ACVPSVPCPL PTQGGFSSCS GGRSSSVRFV STTTSCRTKY

**GN=KRT85: Keratin, type II cuticular Hb5 OS=Homo sapiens**

**From Library (96.8%) and Sequest (89.4%):**

MSCRSYR**ISS GCGVTRNFSS CSAVAPKTGN RCCISAAPYR** GVSCYRGLTG FGSR**SLCNLG SCGPR**IAVGG

FRAGSCGRSF GYR**SGGVCGP SPPCITTVSV NESLLTPLNL EIDPNAQCVK QEEKEQIKSL NSRFAAFIDK**

**VRFLEQQNKL LETKWQFYQN QRCCESNLEP LFSGYIETLR REAECVEADS GRLASELNHV QEVLEGYKKK**

**YEEEVALRAT AENEFVVLKK DVDCAYLRKS DLEANVEALV EESSFLRRLY EEEIRVLQAH ISDTSVIVKM**

**DNSRDLNMDC IIAEIKAQYD DVASRSRAEA ESWYRSKCEE MKATVIR**HGE TLR**RTKEEIN ELNR**MIQR**LT**

**AEIENAKCQR** **AKLEAAVAEA EQQGEAALSD ARCKLAELEG ALQKAKQDMA CLLKEYQEVM NSKLGLDIEI**

**ATYRRLLEGE EHRLCEGVGS VNVCVSSSRG GVSCGGLSYS TTPGRQITSG PSAIGGSITV VAPDSCAPCQ**

**PRSSSFSCGS SR**SVRFA

**GN=KRT86: Keratin, type II cuticular Hb6 OS=Homo sapiens**

**From Library (99.2%) and Sequest (92.4%):**

MTCGSYCGGR **AFSCISACGP RPGRCCITAA PYR**GISCY**RG LTGGFGSHSV CGGFR**AGSCG RSFGYR**SGGV**

**CGPSPPCITT VSVNESLLTP LNLEIDPNAQ CVKQEEKEQI KSLNSRFAAF IDKVRFLEQQ NKLLETKLQF**

**YQNRECCQSN LEPLFEGYIE TLRREAECVE ADSGRLASEL NHVQEVLEGY KKKYEEEVSL RATAENEFVA**

**LKKDVDCAYL RKSDLEANVE ALIQEIDFLR RLYEEEIRVL QSHISDTSVV VKLDNSRDLN MDCIIAEIKA**

**QYDDIVTRSR AEAESWYRSK CEEMKATVIR** HGETLR**RTKE EINELNR**MIQ **RLTAEVENAK CQNSKLEAAV**

**AQSEQQGEAA LSDARCKLAE LEGALQKAKQ DMACLIREYQ EVMNSKLGLD IEIATYRRLL EGEEQRLCEG**

**VGSVNVCVSS SRGGVVCGDL CASTTAPVVS TRVSSVPSNS NVVVGTTNAC APSARVGVCG GSCK**RC
