# Supplementary material for: Sensitive Method for the Confident Identification of Genetically Variant Peptides in Human Hair Keratin
Source: J Forensic Sci. 2019 Oct 31;65(2):406–20. doi: 10.1111/1556-4029.14229 (PMC7064992; doi:10.1111/1556-4029.14229)
Supplement: Supplementary file 3 — Appendix S3. GVP panel analyses in all ten fractions by the direct method. [file JFO-65-406-s003.docx]

SUPPLEMENTARY DOCUMENT S3—*GVP panel analyses in all ten fractions by the direct method.*

# Example GVP Panel Analysis

(D_LG_F1_TO_F10_R1)

Outlines:

- GVP Panel Analysis Overall
  - Left: Names and sequences of total 14 GVPs and non-variants
  - Right: Highest abundance in each fraction of “D_LG_F1_TO_F10_R1”
- High Abundance GVP pairs
  - Type I example
  - Type II example
- Check Low Abundance GVPs
  - Check spectral match
    - MS Search: search inquiry spectrum against library spectra
  - Check MS1 peak
    - Xcalibur Qual Brower
- Check Low Abundance Regular Forms (if applicable)
  - Check spectral match
    - MS Search: search inquiry spectrum against library spectra
  - Check MS1 peak
    - Xcalibur Qual Brower
- Summary Sheet


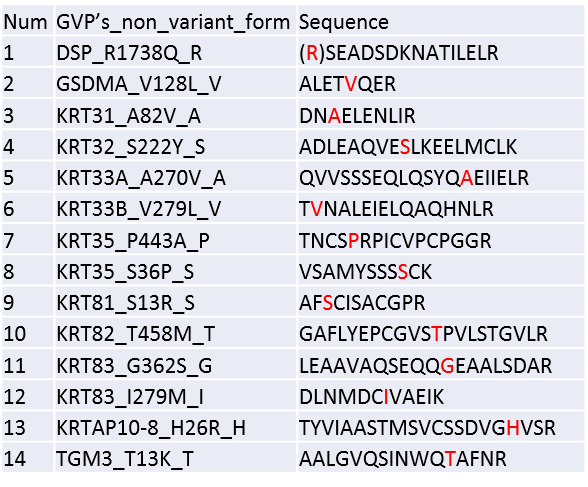

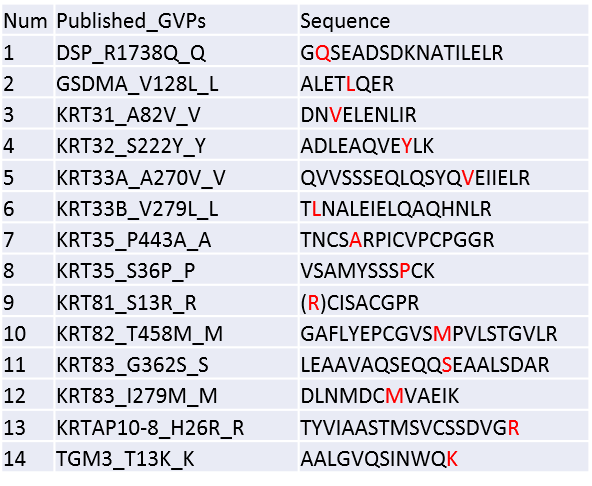

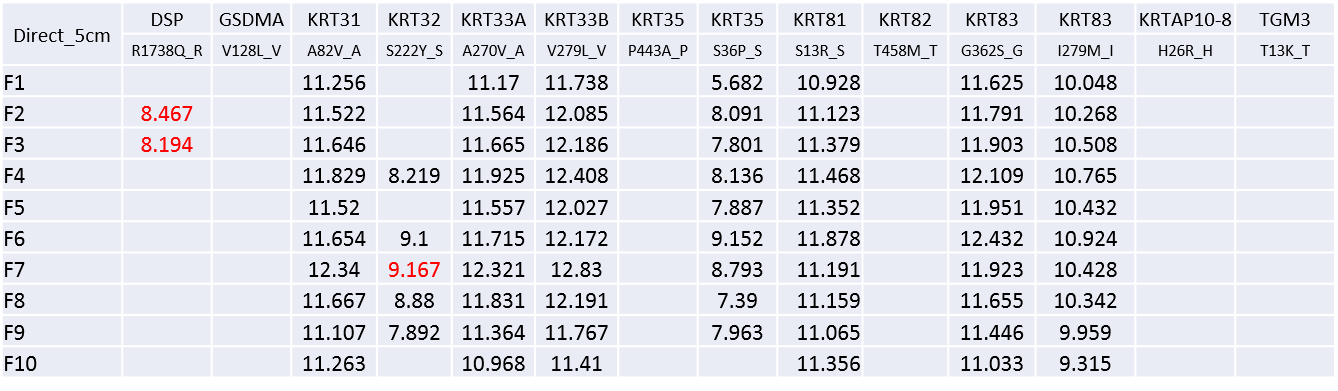

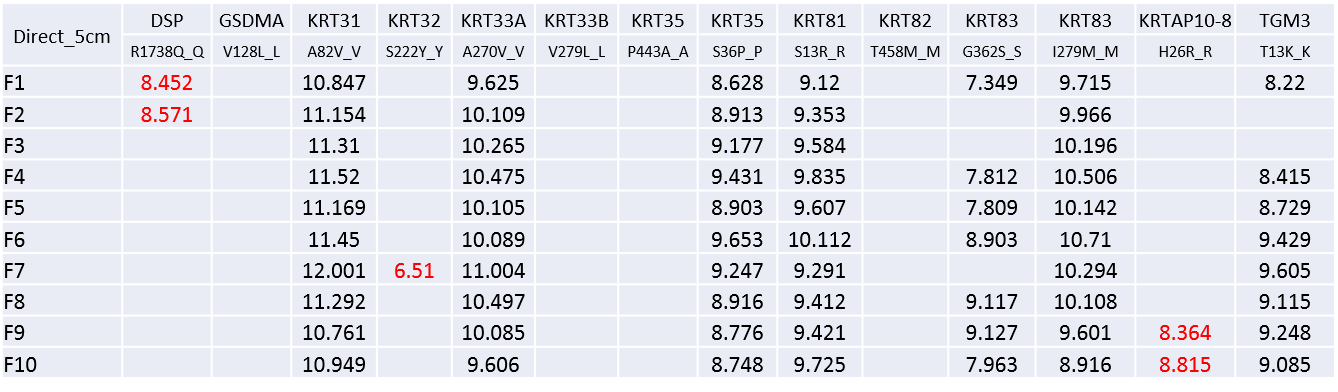


Type II example

Type I example

Type I example

Type II example

**Variant Highest log10 Abundance**

**Non-variant Highest log10 Abundance**

1

2

3

4

5

6

7

8

9

10

11

12

13

14

1

2

3

4

5

6

7

8

9

10

11

12

13

14

Checked

Checked

Checked

Checked

Checked

# High Abundance GVP pairs

Type I example

Type I example, also shown in Figure 2A

14


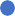

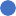

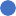

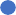

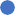

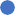

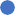

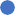

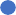

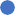


13

log10 (Abund)

12

11

10

| Direct_5cm | KRT33A | RT | MF |
| --- | --- | --- | --- |
|  | A270V_V QVVSSSEQLQSYQVEIIELR/3_0 |  |  |
| F1 | 9.625 | 162.2 | 935 |
| F2 | 10.109 | 161.7 | 938 |
| F3 | 10.265 | 161.1 | 942 |
| F4 | 10.475 | 160.5 | 938 |
| F5 | 10.105 | 160.4 | 869 |
| F6 | 10.089 | 155.1 | 607 |
| F7 | 11.004 | 157.7 | 803 |
| F8 | 10.497 | 156.8 | 813 |
| F9 | 10.085 | 156.7 | 906 |
| F10 | 9.606 | 155.3 | 792 |
|  |  |  |  |
| Direct_5cm | KRT33A | RT | MF |
|  | A270V_A QVVSSSEQLQSYQAEIIELR/3_0 |  |  |
| F1 | 11.17 | 160.5 | 902 |
| F2 | 11.564 | 160.0 | 897 |
| F3 | 11.665 | 159.2 | 796 |
| F4 | 11.925 | 158.7 | 892 |
| F5 | 11.557 | 158.6 | 904 |
| F6 | 11.715 | 152.5 | 900 |
| F7 | 12.321 | 155.1 | 900 |
| F8 | 11.831 | 154.2 | 903 |
| F9 | 11.364 | 154.3 | 908 |
| F10 | 10.968 | 152.8 | 910 |

9

8

1 2 3 4 5 6 7 8 9 10


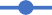
KRT33A A270V_V QVVSSSEQLQSYQVEIIELR/3_0 KRT33A A270V_A QVVSSSEQLQSYQAEIIELR/3_0

Fractions

Type II example

| Direct_5cm | KRT83 | RT | MF |
| --- | --- | --- | --- |
|  | I279M_M DLNMDCMVAEIK/2_3/4,M,Oxidation/6,C,Carbamidomethyl/7,M,Oxidation |  |  |
| F1 | 9.715 | 104.8 | 813 |
| F2 | 9.966 | 103.5 | 802 |
| F3 | 10.196 | 103.3 | 829 |
| F4 | 10.506 | 102.1 | 847 |
| F5 | 10.142 | 101.2 | 819 |
| F6 | 10.71 | 91.6 | 792 |
| F7 | 10.294 | 95.5 | 853 |
| F8 | 10.108 | 93.5 | 807 |
| F9 | 9.601 | 94.6 | 873 |
| F10 | 8.916 | 93.1 | 349 |
|  |  |  |  |
| Direct_5cm | KRT83 | RT | MF |
|  | I279M_I DLNMDCIVAEIK/2_2/4,M,Oxidation/6,C,Carbamidomethyl |  |  |
| F1 | 10.048 | 137.2 | 887 |
| F2 | 10.268 | 135.7 | 909 |
| F3 | 10.508 | 134.1 | 865 |
| F4 | 10.765 | 134.2 | 891 |
| F5 | 10.432 | 134.2 | 818 |
| F6 | 10.924 | 124.8 | 921 |
| F7 | 10.428 | 128.6 | 895 |
| F8 | 10.342 | 127.2 | 885 |
| F9 | 9.959 | 128.4 | 931 |
| F10 | 9.315 | 125.8 | 887 |

Type II example, also shown in Figure 2B

12


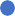

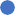

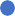

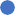

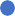

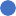

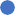

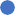

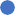

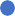


11

10

log10 (Abund)

9

8

1 2 3 4 5 6 7 8 9 10

KRT83 I279M_M


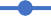
DLNMDCMVAEIK/2_3/4,M,Oxidation/6,C,Carbamidomethyl/7,M,Oxidation KRT83 I279M_I DLNMDCIVAEIK/2_2/4,M,Oxidation/6,C,Carbamidomethyl

Fractions

# Check Low Abundance GVPs

KRT32_S222Y_Y: ADLEAQVEYLK


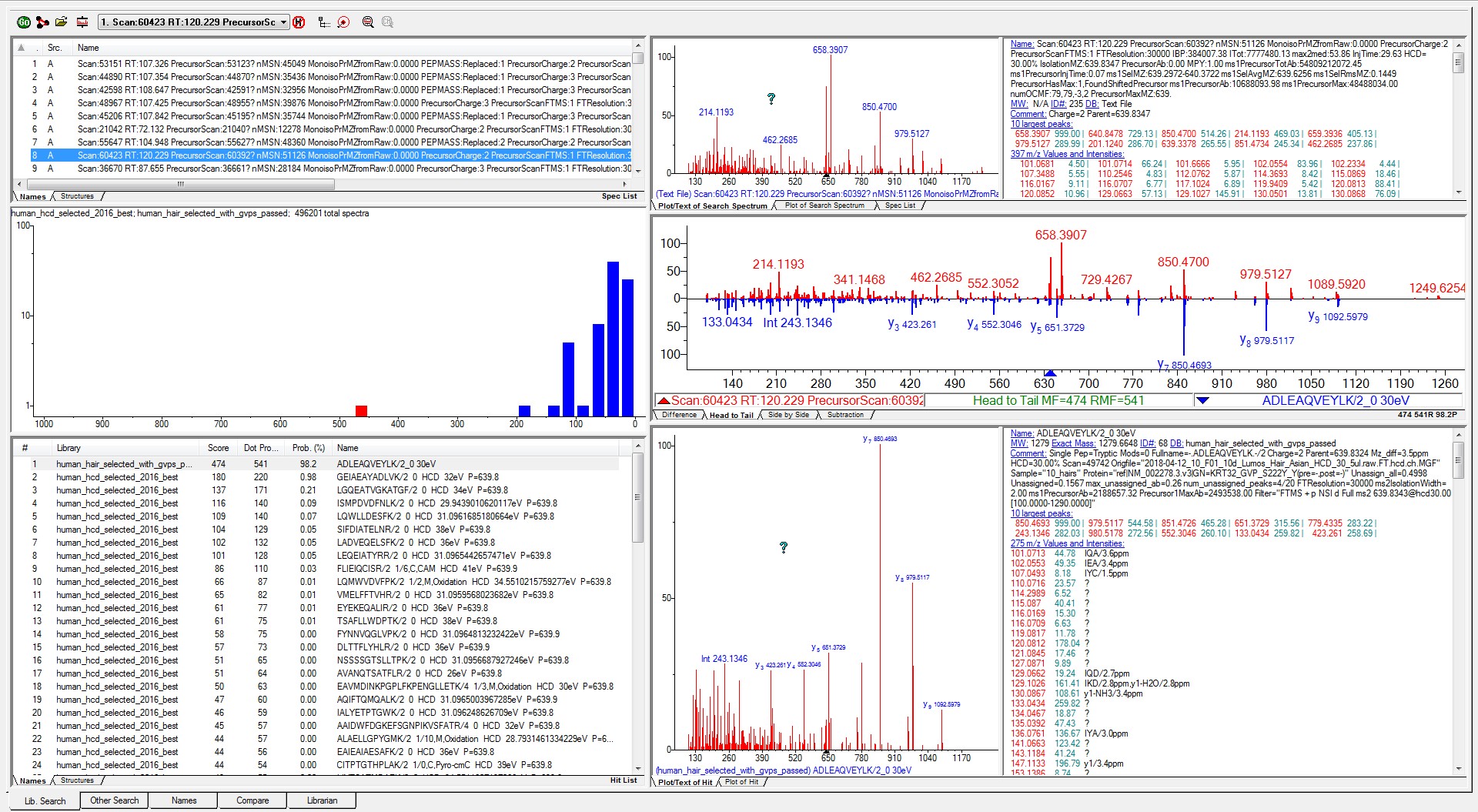
in Fraction 7 ‘hold’ to be confirmed


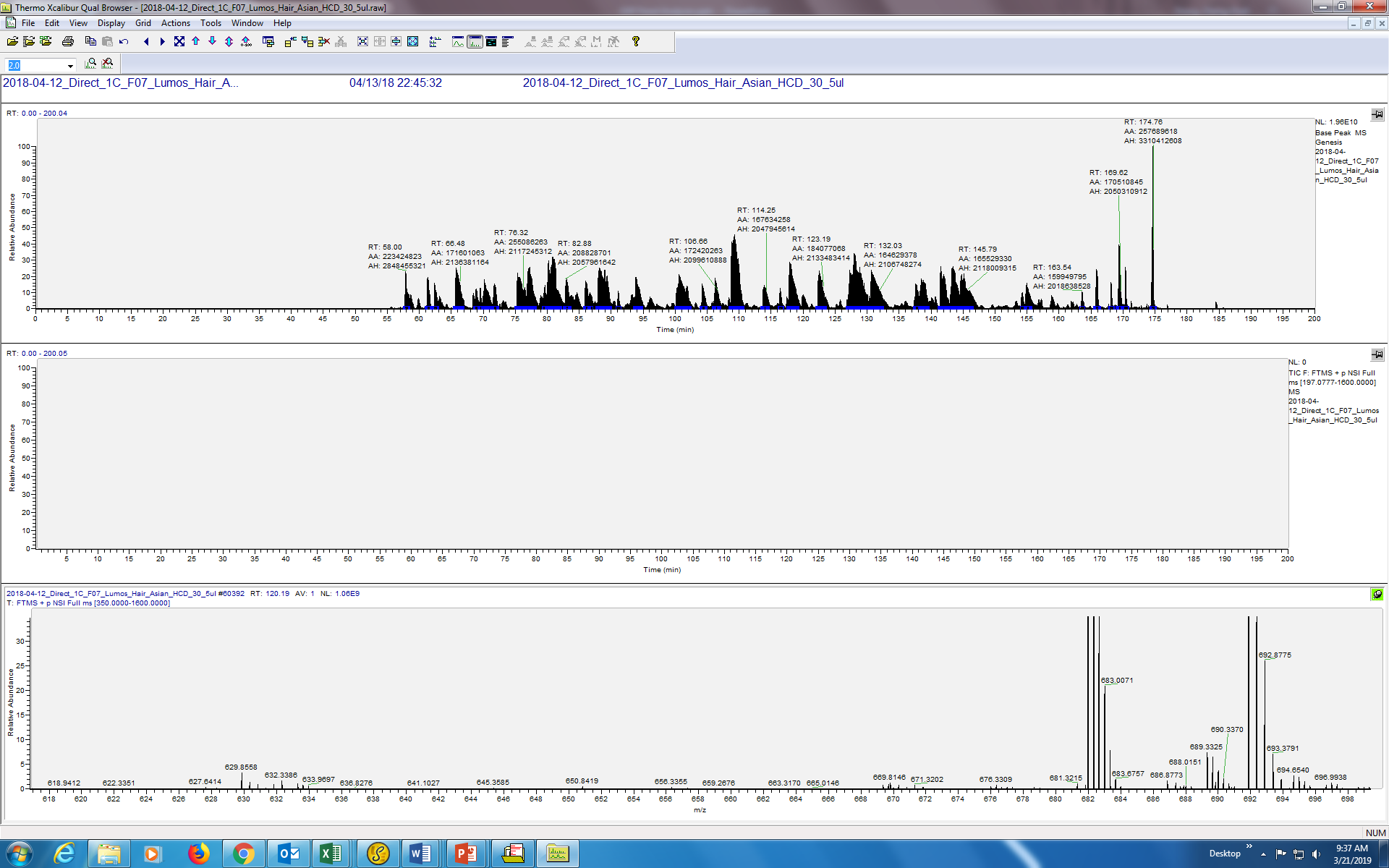


No MS1 Peak at 639.8347

DSP_R1738Q_Q: GQSEADSDKNATILELR


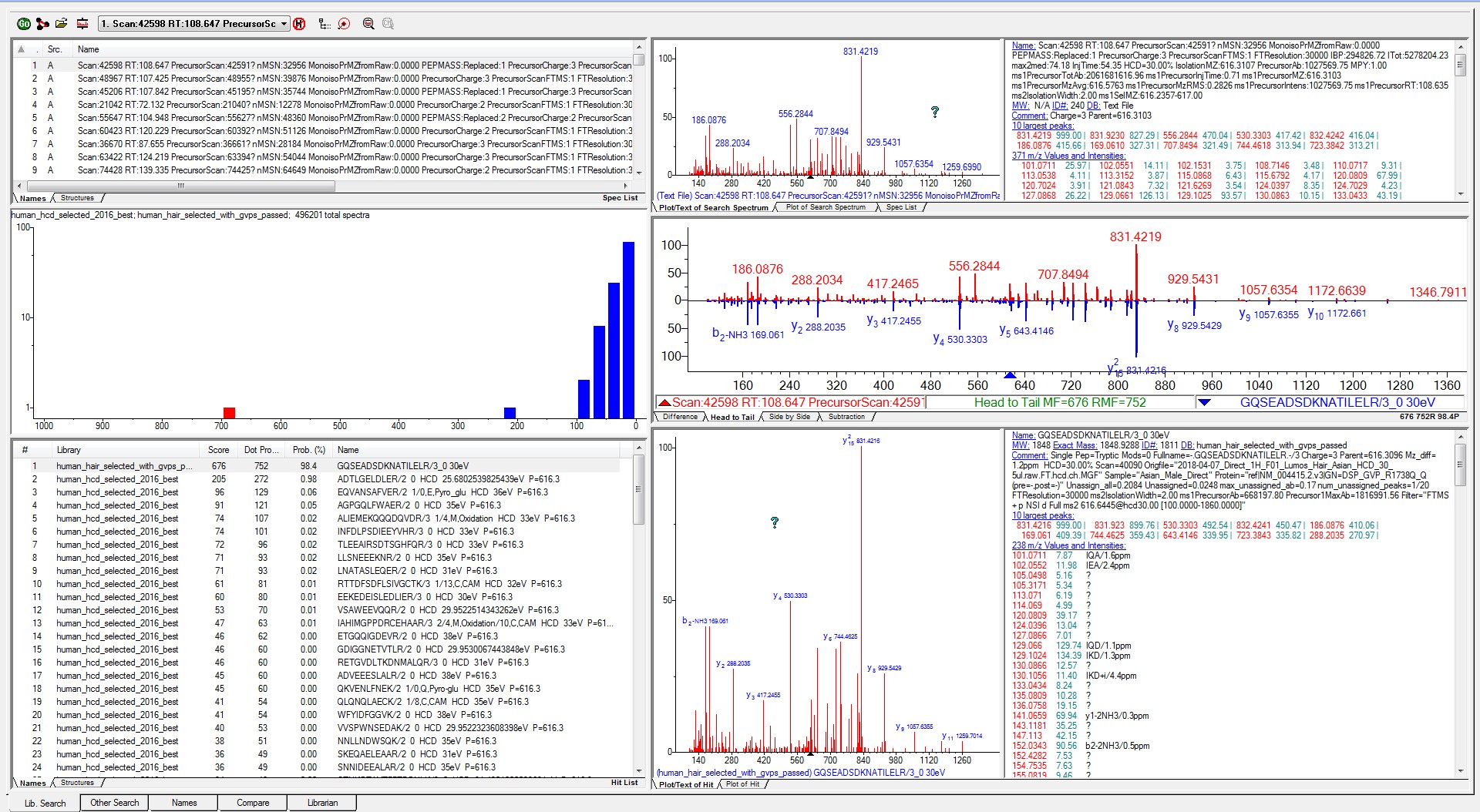
in Fraction 1 confirmed


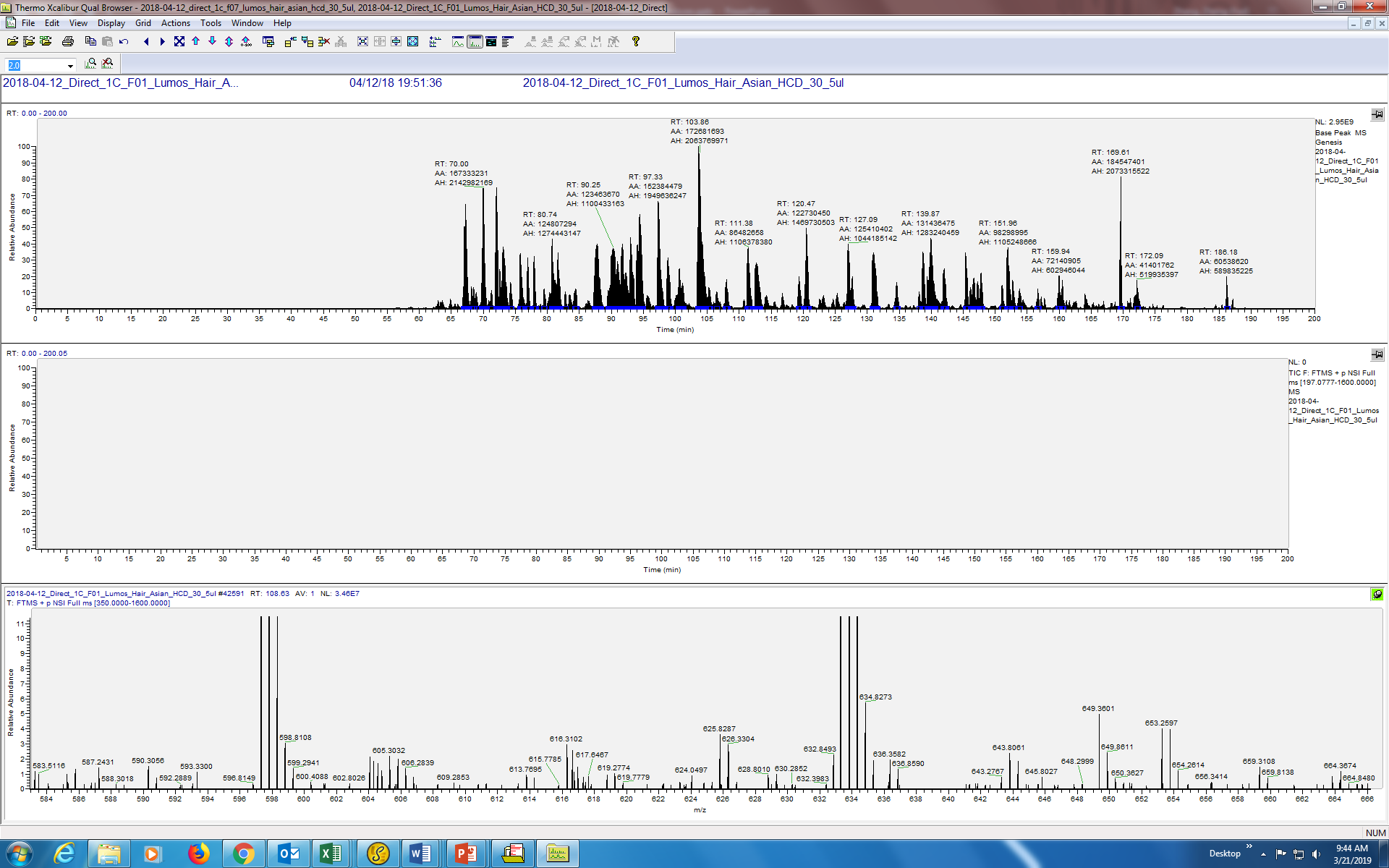


MS1 Peak

DSP_R1738Q_Q: GQSEADSDKNATILELR


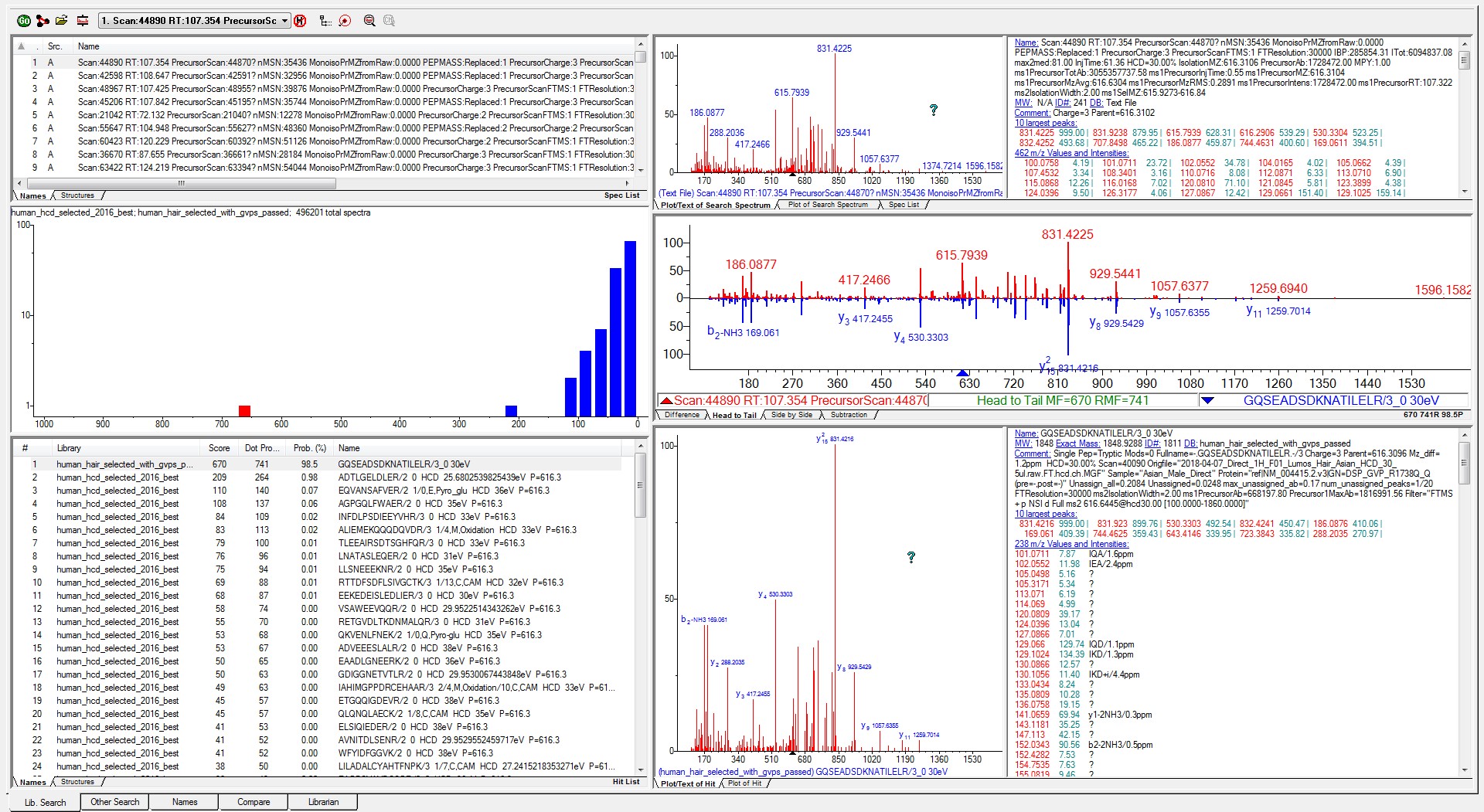
in Fraction 2 confirmed


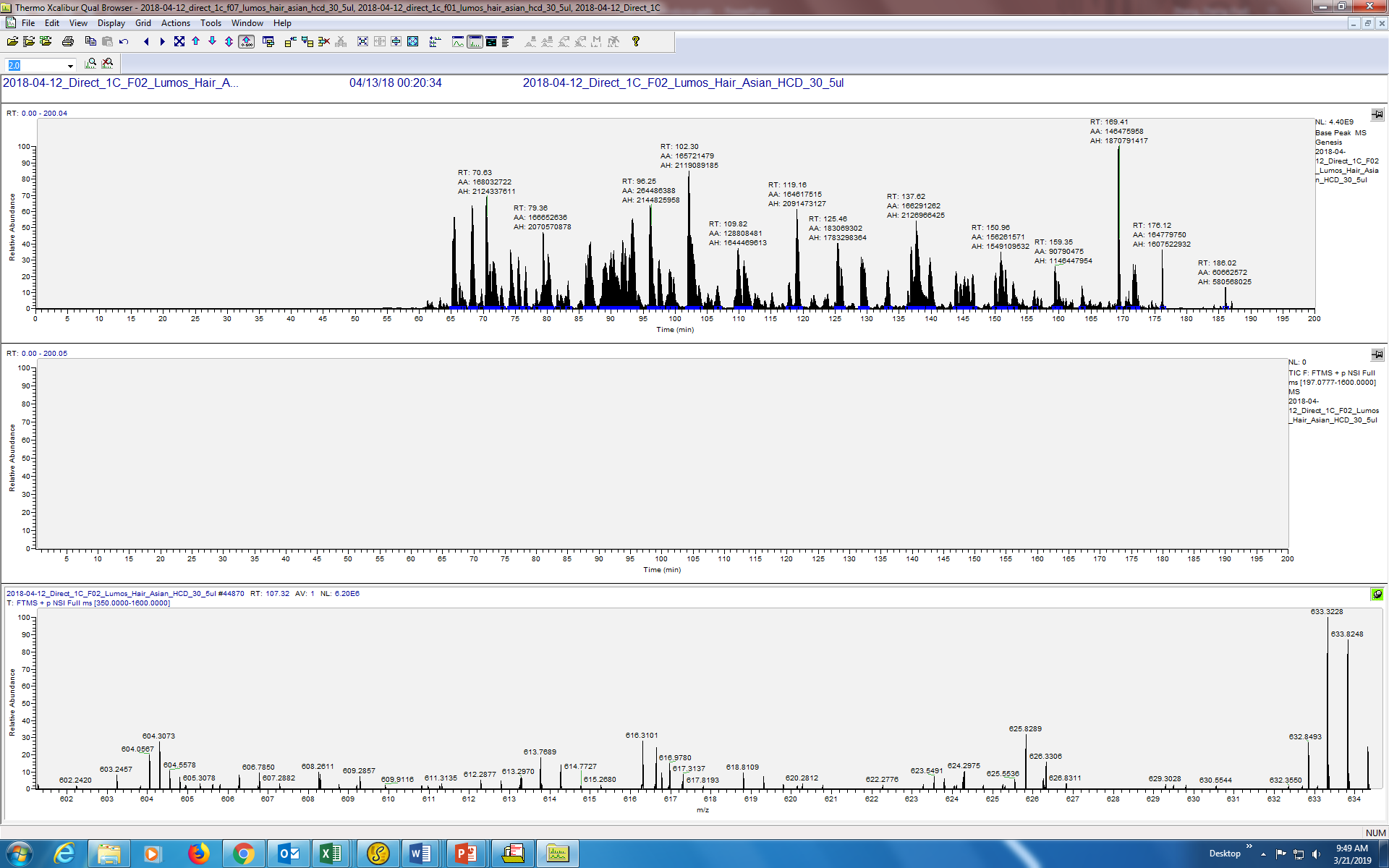


MS1 Peak

KRTAP10-8_H26R_R: TYVIAASTMSVCSSDVGR


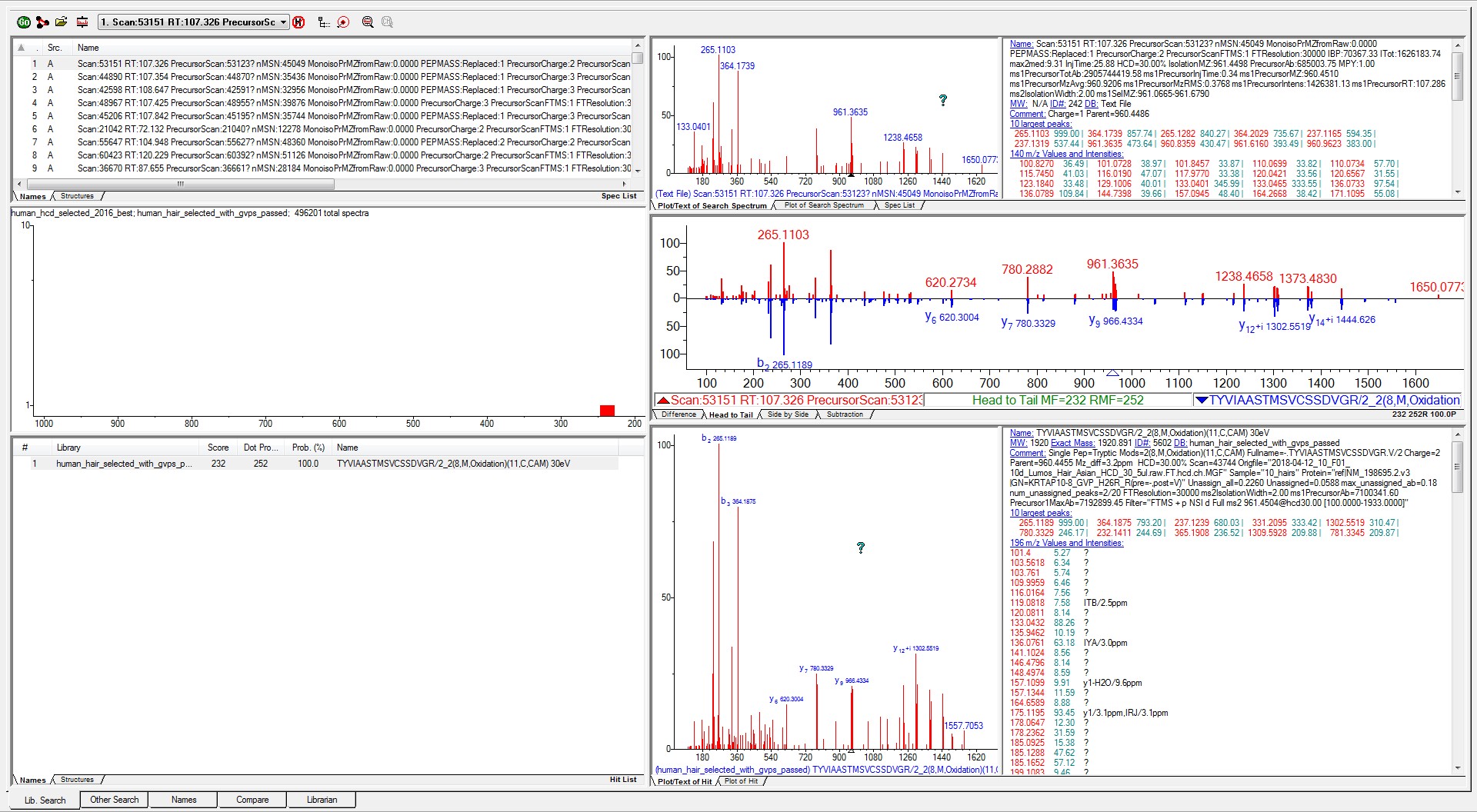
in Fraction 9 confirmed


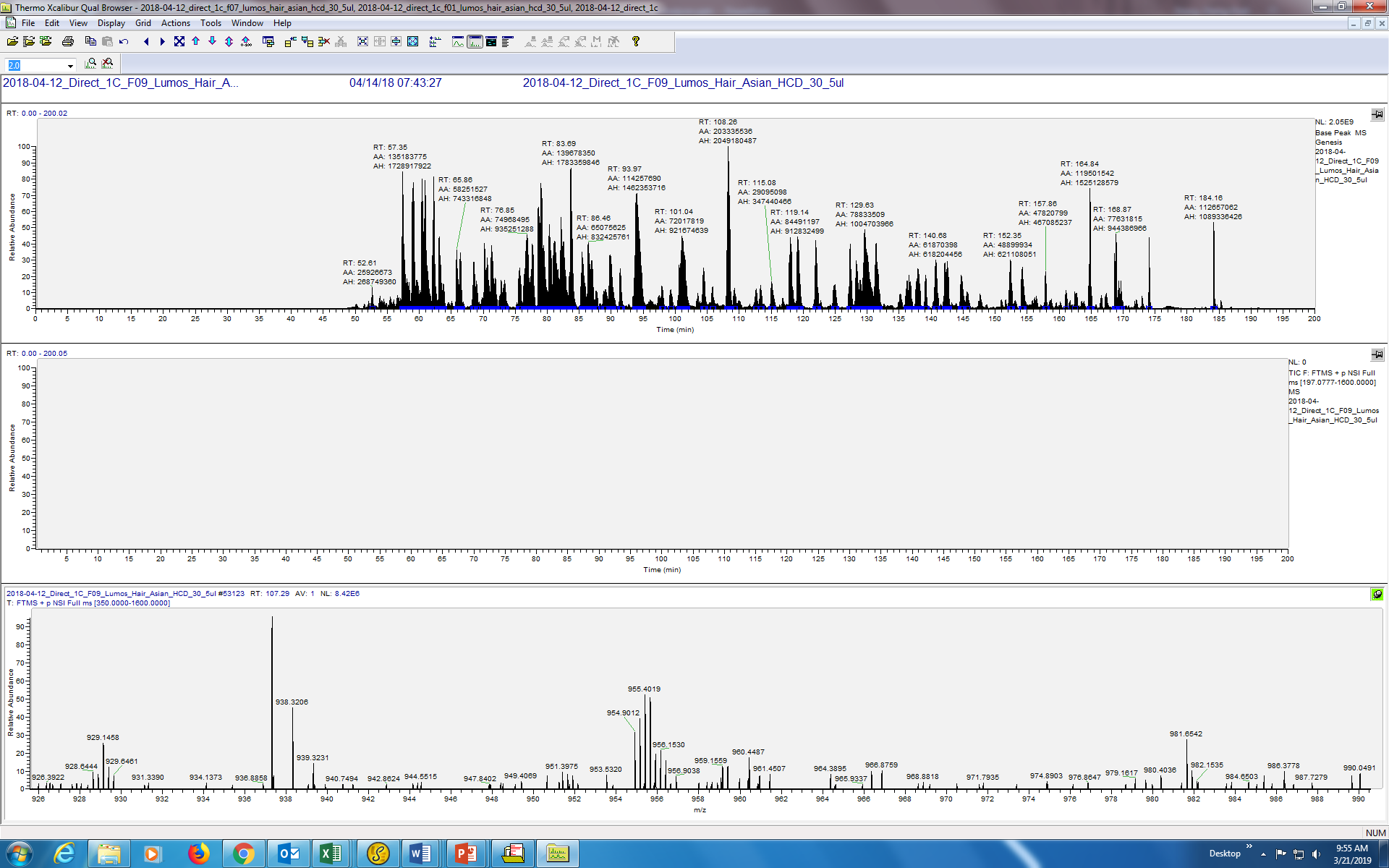


MS1 Peak


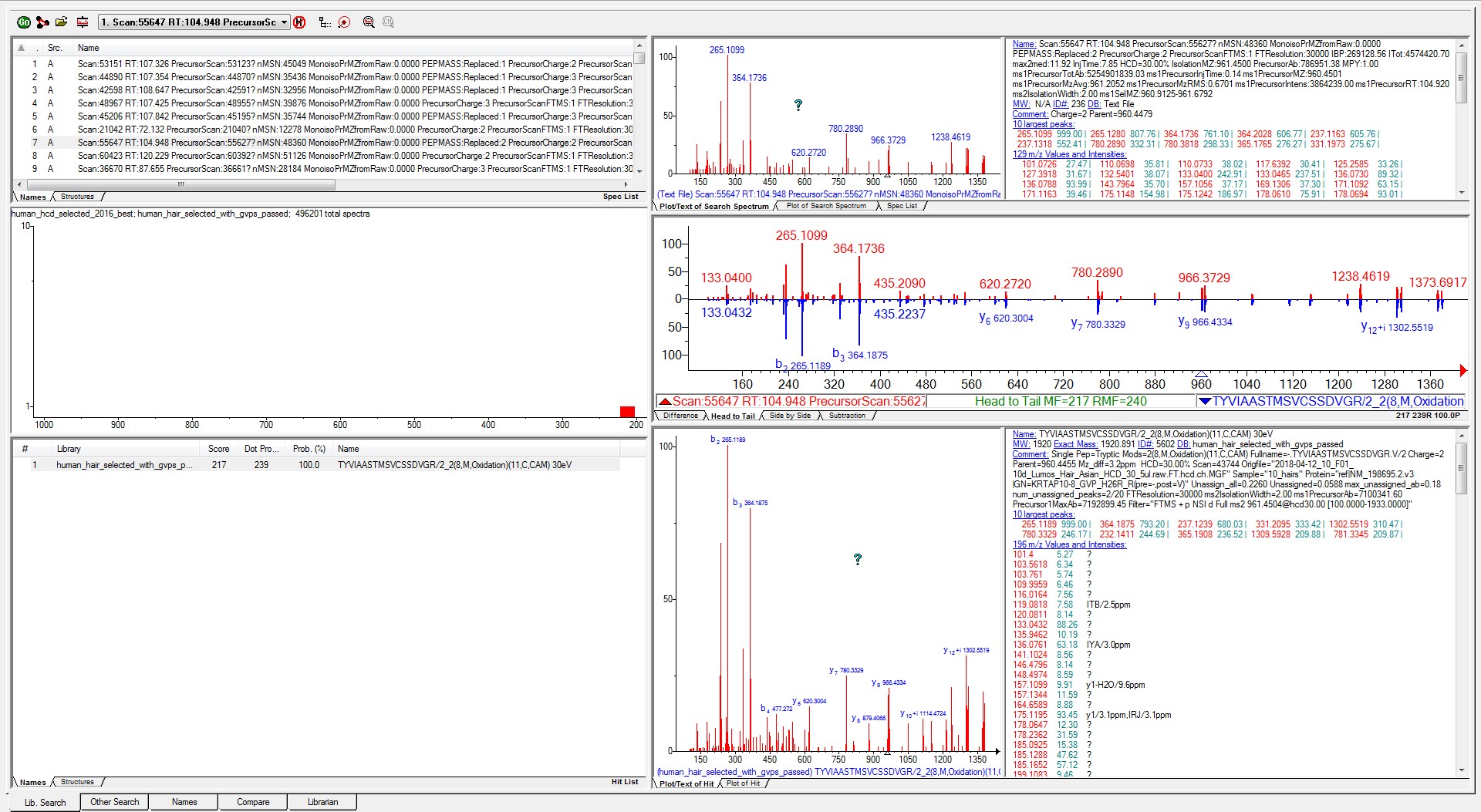
KRTAP10-8_H26R_R: TYVIAASTMSVCSSDVGR

in Fraction 10 confirmed


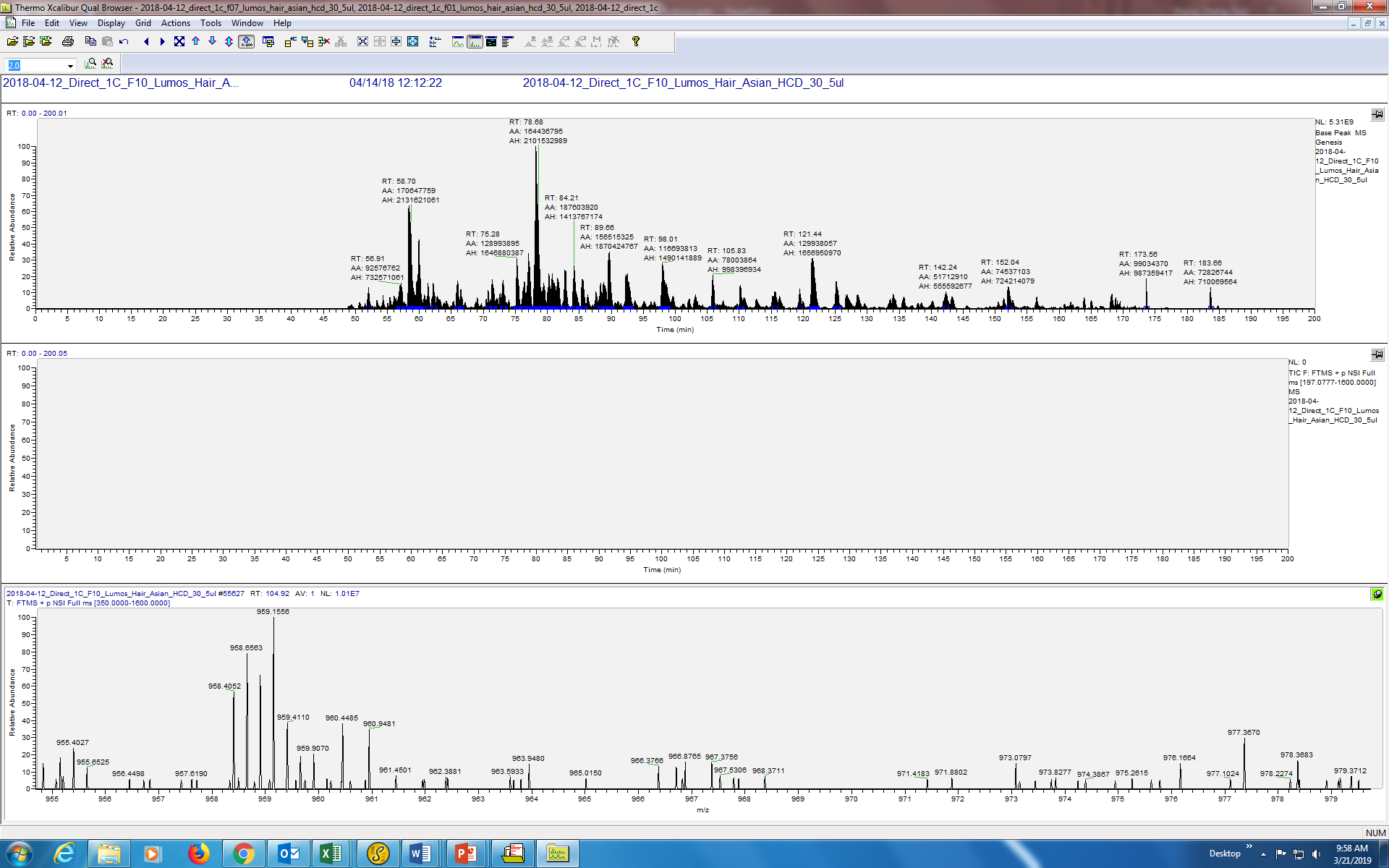


MS1 Peak

# Check Low Abundance Regular Forms

(if applicable)

DSP_R1738Q_Q: (R)SEADSDKNATILELR


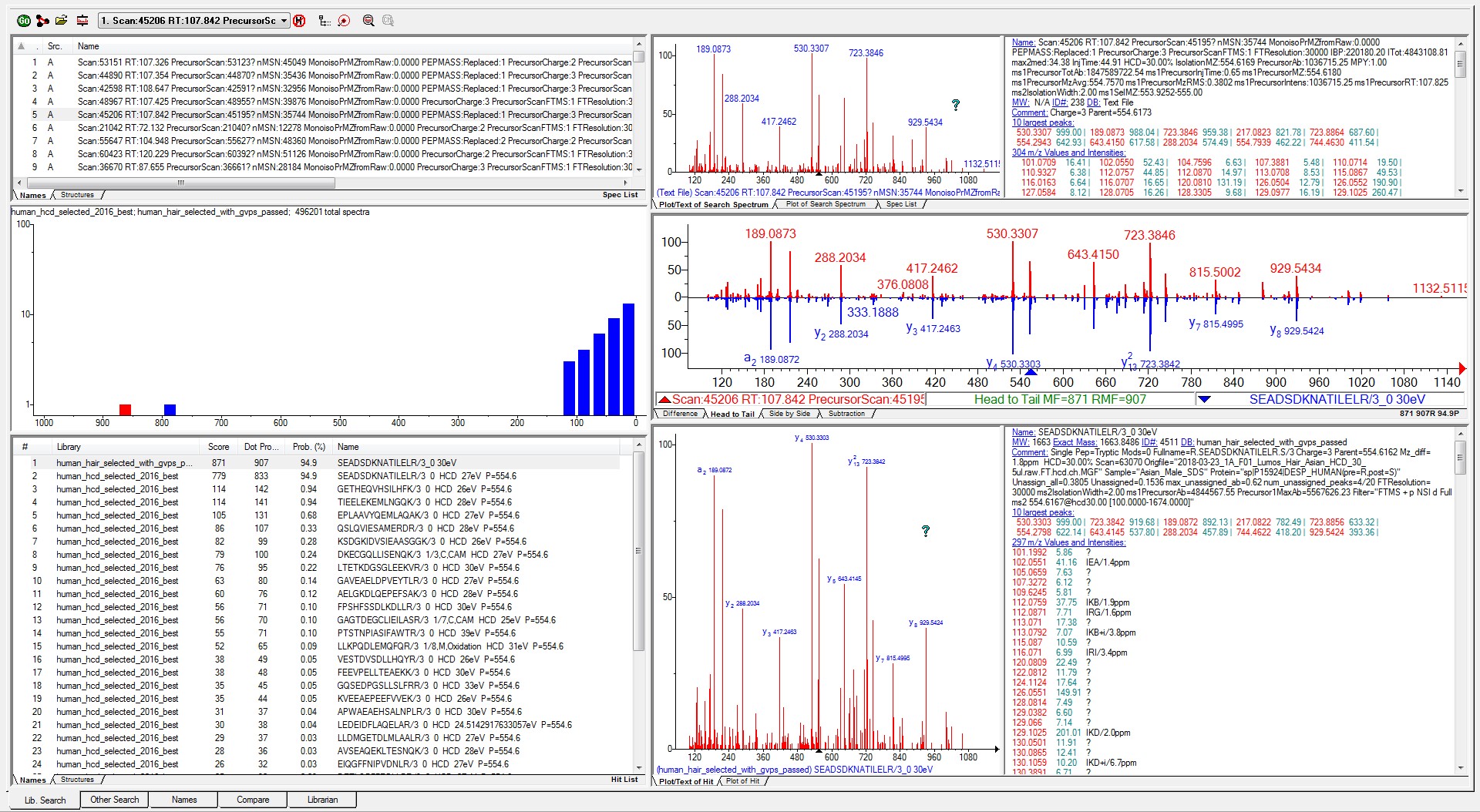
in Fraction 2 confirmed


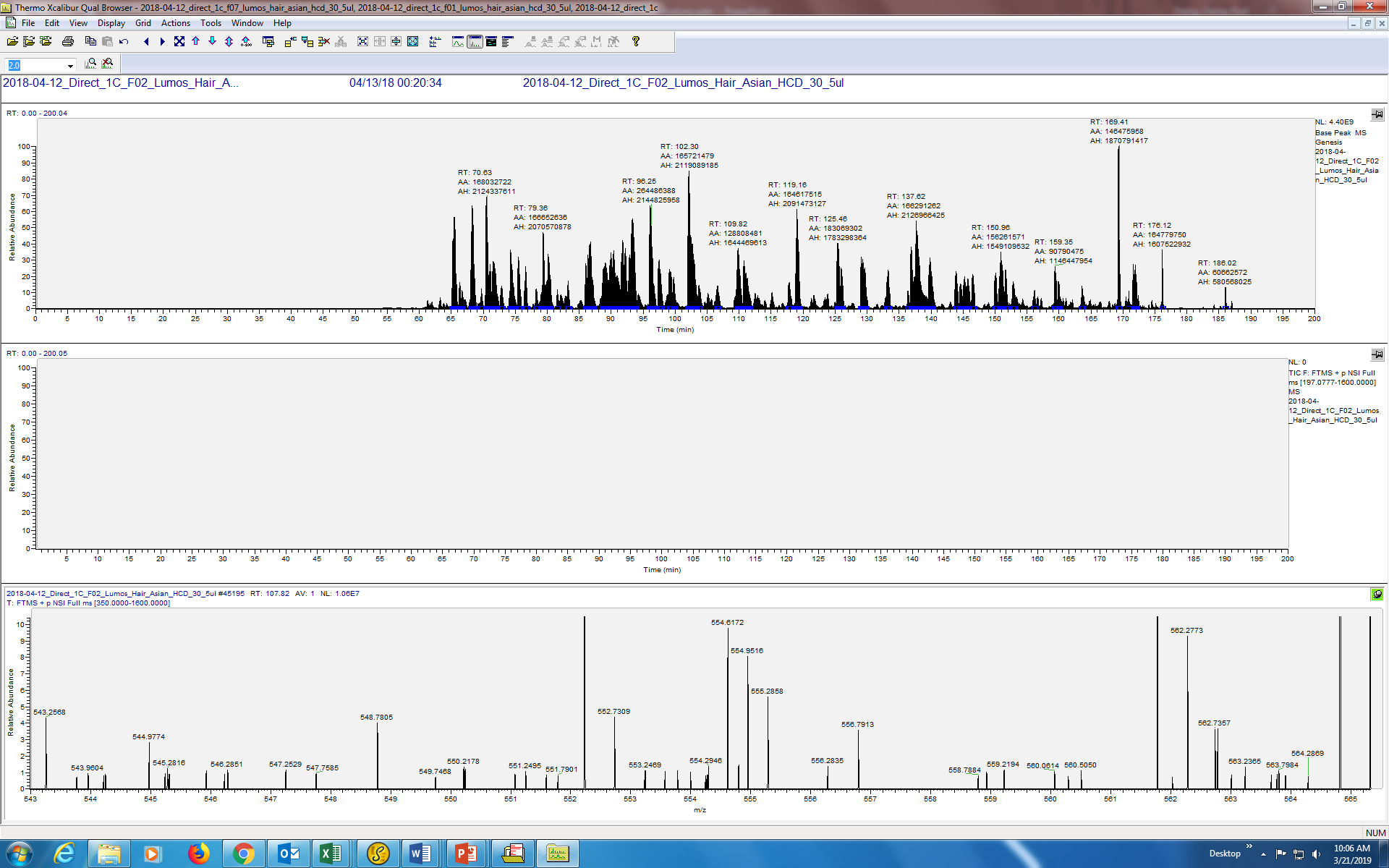


MS1 Peak


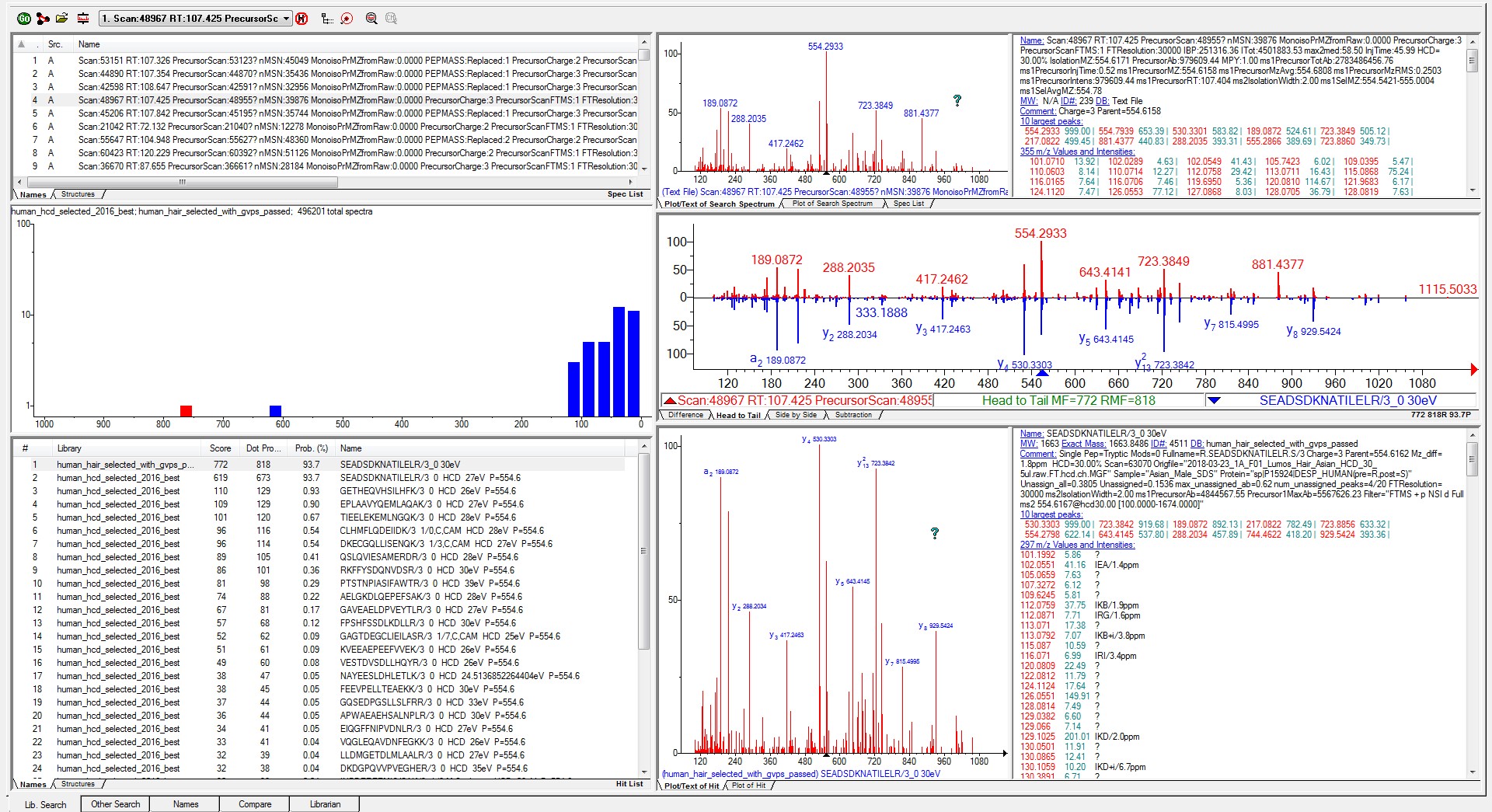
DSP_R1738Q_Q: (R)SEADSDKNATILELR

in Fraction 3 confirmed


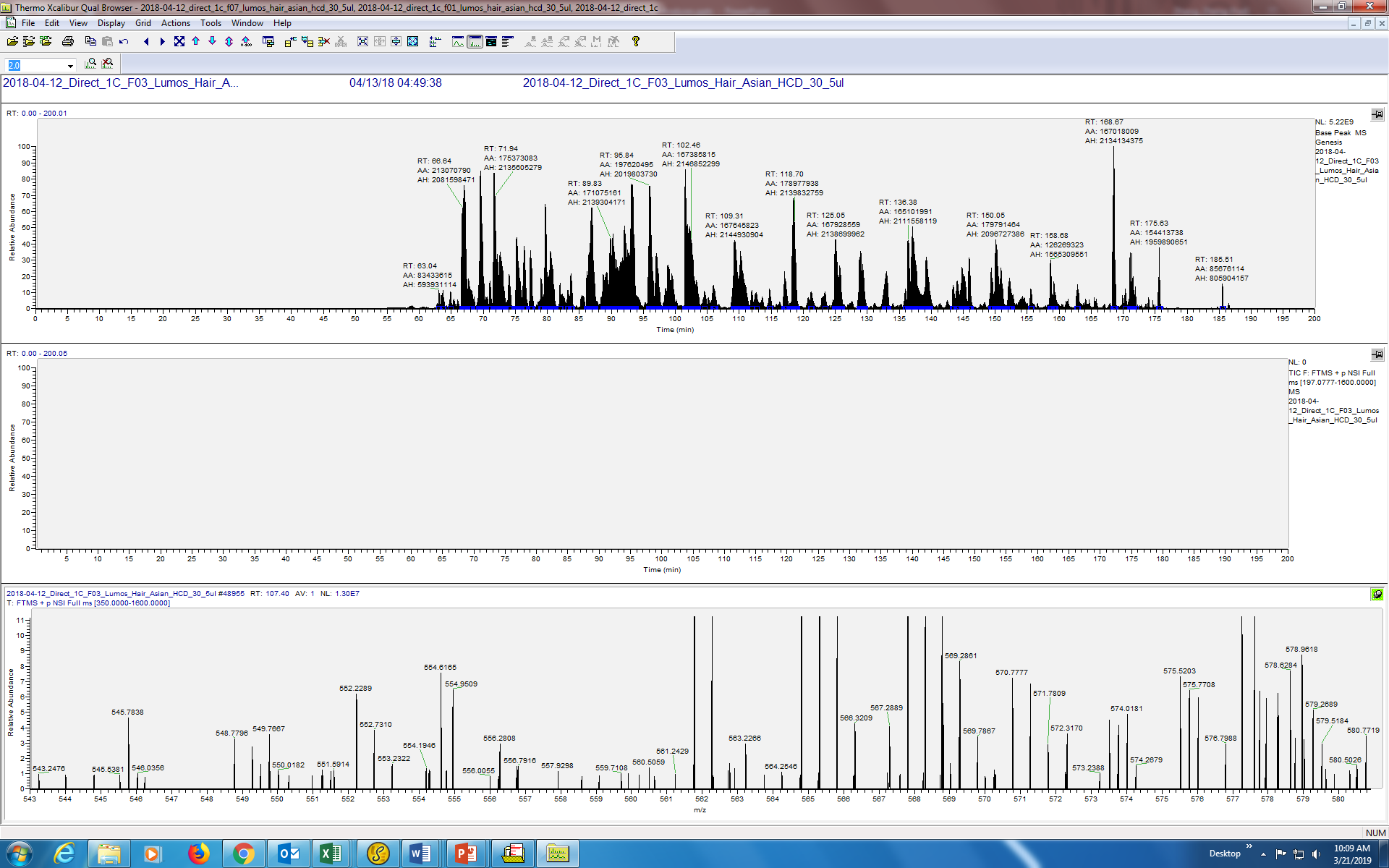


MS1 Peak

KRT32_S222Y_S: ADLEAQVE(S)LKEELMCLK


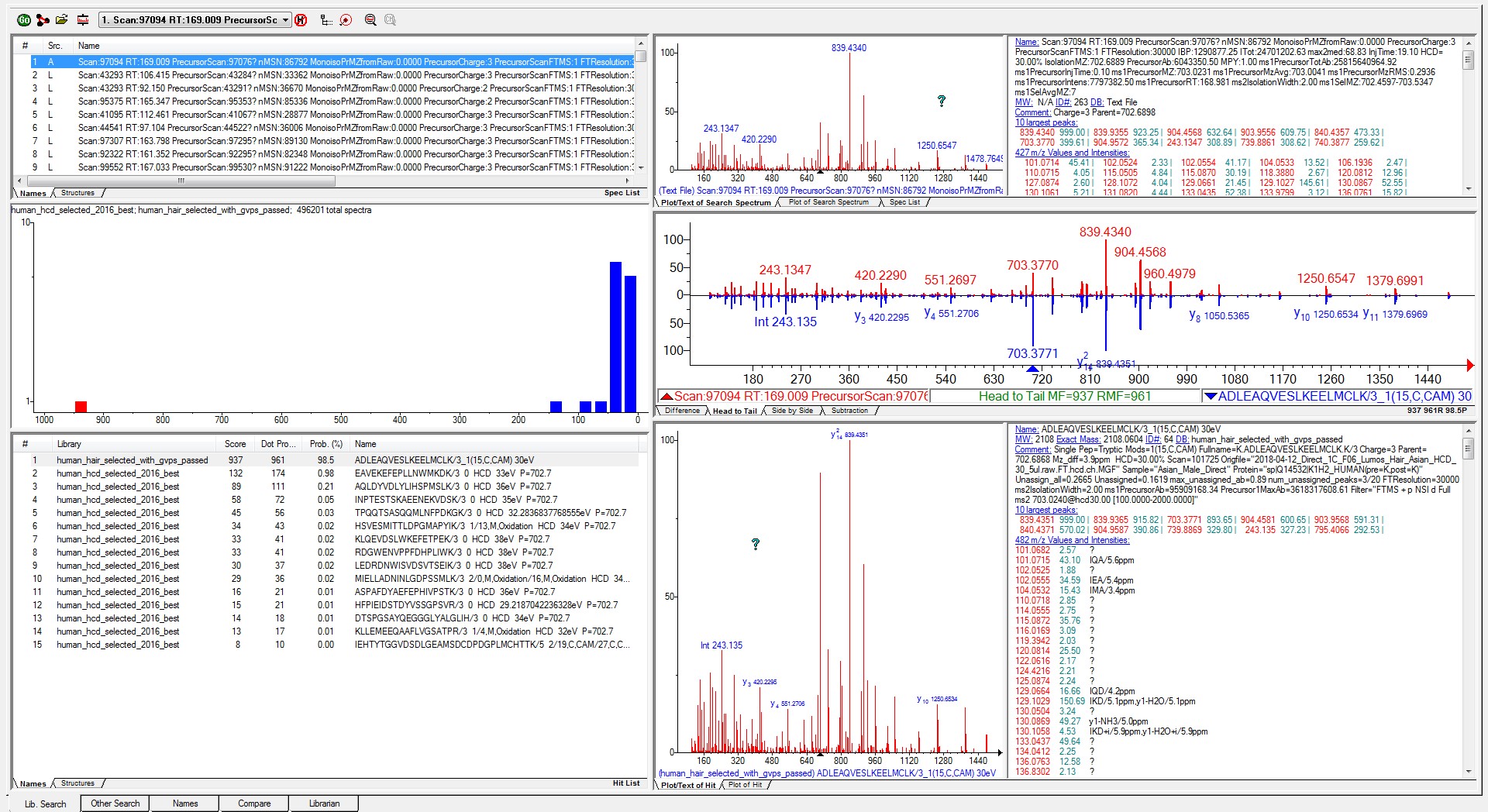
in Fraction 7 confirmed

| 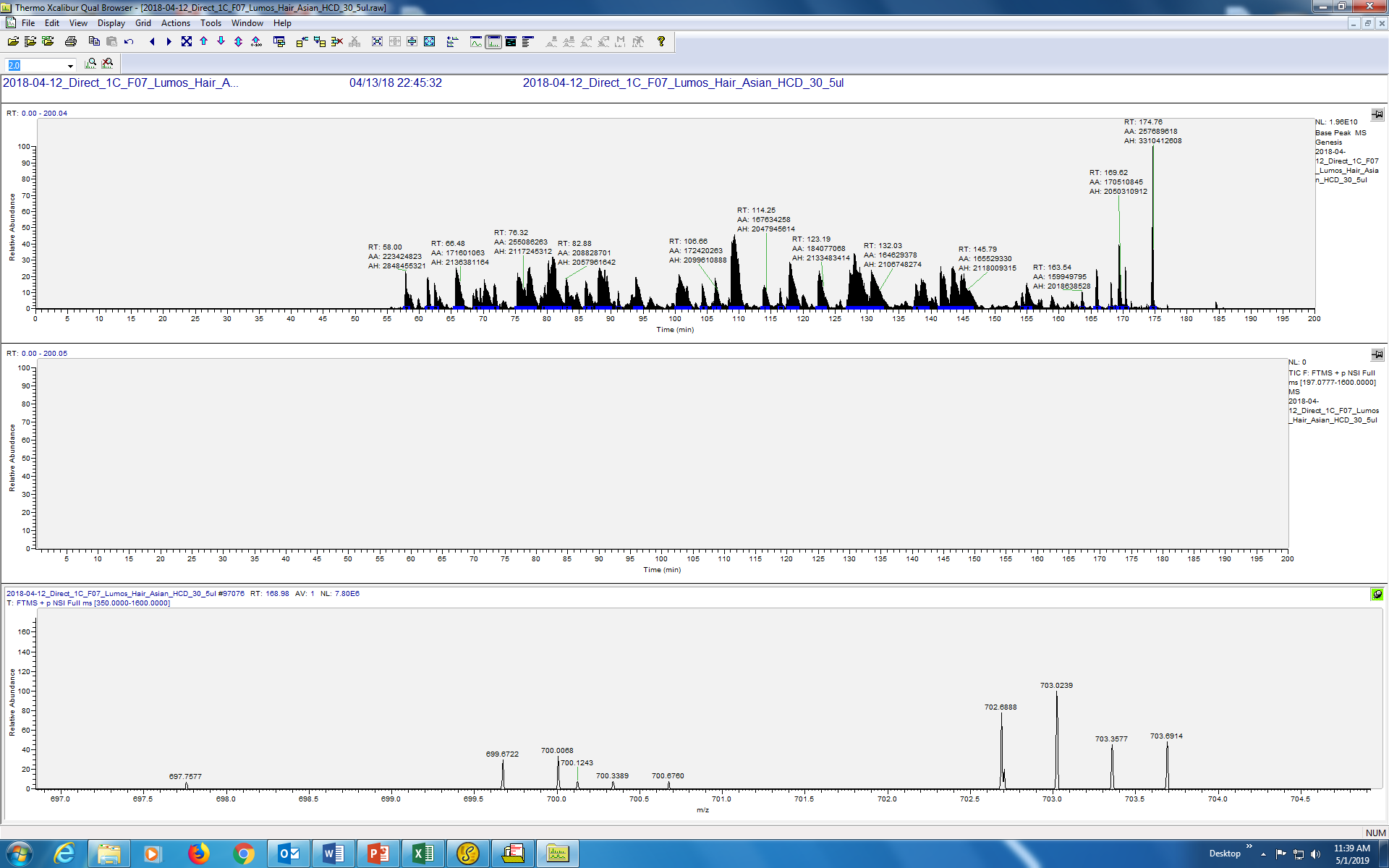 MS1 Peak |  |  |  |  |  |  |  |  |  |  |  |  |  |
| --- | --- | --- | --- | --- | --- | --- | --- | --- | --- | --- | --- | --- | --- |

**GVP sites are summarized from all 10 fractions:**

| **Asian_1hair_5cm** | **DSP** | **GSDMA** | **KRT31** | **KRT32** | **KRT33A** | **KRT33B** | **KRT35** | **KRT35** | **KRT81** | **KRT82** | **KRT83** | **KRT83** | **KRTAP10-8** | **TGM3** |
| --- | --- | --- | --- | --- | --- | --- | --- | --- | --- | --- | --- | --- | --- | --- |
|  | **R1738Q_Q** | **V128L_L** | **A82V_V** | **S222Y_Y** | **A270V_V** | **V279L_L** | **P443A_A** | **S36P_P** | **S13R_R** | **T458M_M** | **G362S_S** | **I279M_M** | **H26R_R** | **T13K_K** |
| **D_LG_F1_TO_F10_R1** | **X** |  | **X** | ?* | **X** |  |  | **X** | **X** |  | **X** | **X** | **X** | **X** |

***note for “?”: It means we cannot confirm its identification at this time with a borderline intensity and lack of MS1 peak. However, some of the major peaks still match well and it showed up from the expected fraction. For such case, we put “hold” to be confirmed.**

- Analyses above led to several general findings:
  - High abundance GVP analysis is very convincing –
    - with its regular non-variant form presenting in all 10 fractions
    - with convincing nistms_metrics information:
      - Abundance (log10)
      - Match Factor (MF)
      - Retention Time (RT)
  - Low abundance GVP analysis is harder, but confidence can be increased by at least one of the following –
    - from expected gel bands (based on molecular weight of its protein)
    - with the presence of its regular non-variant form
    - with convincing nistms_metrics information:
      - MF
      - RT
      - MS1 Peak
