# Supplementary material for: Sensitive Method for the Confident Identification of Genetically Variant Peptides in Human Hair Keratin
Source: J Forensic Sci. 2019 Oct 31;65(2):406–20. doi: 10.1111/1556-4029.14229 (PMC7064992; doi:10.1111/1556-4029.14229)
Supplement: Supplementary file 4 — Appendix S4. Histograms of the distribution of all DeltaMass values in three methods. [file JFO-65-406-s004.docx]

SUPPLEMENTARY DOCUMENT S4—*Histograms of the distribution of all DeltaMass values in three methods.* *Distribution of DeltaMass values obtained from hybrid search identifications of hair-derived peptides (hair shaft length of 5 cm) extracted by the cleavable surfactant (red), NaOH+SDS (green), and direct (blue) method above a spectral match score threshold of 500. The major labeled peaks in each panel are correspond to those in Table 5.*
